# Structural Analysis of Lignin-Based Furan Resin

**DOI:** 10.3390/ma15010350

**Published:** 2022-01-04

**Authors:** Xuhai Zhu, Bardo Bruijnaers, Tainise V. Lourençon, Mikhail Balakshin

**Affiliations:** 1 Department of Bioproducts and Biosystems, School of Chemical Engineering, Aalto University, Vuorimiehentie 1, 02150 Espoo, Finland; tainise@gmail.com; 2 Cellulose-Resin Composite Group, Nemho R&D Group, Trespa International B.V., Wetering 20, 6002 SM Weert, The Netherlands; b.bruijnaers@nemho.com

**Keywords:** lignin, furfuryl alcohol, bio-based resin, structural analysis, NMR

## Abstract

The global “carbon emission peak” and “carbon neutrality” strategic goals promote us to replace current petroleum-based resin products with biomass-based resins. The use of technical lignins and hemicellulose-derived furfuryl alcohol in the production of biomass-based resins are among the most promising ways. Deep understanding of the resulting resin structure is a prerequisite for the optimization of biomass-based resins. Herein, a semiquantitative 2D HSQC NMR technique supplemented by the quantitative ^31^P NMR and methoxyl group wet chemistry analysis were employed for the structural elucidation of softwood kraft lignin-based furfuryl alcohol resin (LFA). The LFA was fractionated into water-insoluble (LFA-I) and soluble (LFA-S) parts. The analysis of methoxyl groups showed that the amount of lignin was 85 wt% and 44 wt% in LFA-I and LFA-S fractions, respectively. The HSQC spectra revealed the high diversity of linkages formed between lignin and poly FA (pFA). The HSQC and ^31^P results indicated the formation of new condensed structures, particularly at the 5-position of the aromatic ring. Esterification reactions between carboxyl groups of lignin and hydroxyl groups of pFA could also occur. Furthermore, it was suggested that lignin phenolic hydroxyl oxygen could attack an opened furan ring to form several aryl ethers structures. Therefore, the LFA resin was produced through crosslinking between lignin fragments and pFA chains.

## 1. Introduction

There is a strong demand to replace current petroleum-based products with environmentally friendly and sustainable alternatives. Lignin-based bioresin, as a 100% renewable product available from lignocellulosic biomass, is of particular interest in this regard. Lignin is the main aromatic biopolymer and can be recovered in large quantities during the commercial production of pulps and cellulosic ethanol [1]; e.g., the global operation of Kraft pulp mills in 2018 resulted in the extraction of about 265 Ktons of lignin, a part of which (5–15%) can be isolated from the black liquors as market kraft lignin (KL) products [2]. Hemicellulose, one of the three major components of biomass, can be converted to various furan chemicals, such as furfural or furfuryl alcohol [3,4]. Therefore, the production of bioresin with KL and hemicellulose-derived furan monomers is attractive for producing a high-value polymer from biomass.

In general, this kind of bioresin can be classified as furan resin, which refers to polymers made with various furan monomers, such as furfural, furfuryl alcohol, or their copolymers with urea, phenol, formaldehyde, lignin etc. [5,6,7]. Over the past few years, lignin-based furfuryl alcohol (LFA) resins have gained a lot of attention from researchers given that it confers structural stability and good water resistance to adhesives [8,9,10]. For example, Gardziella et al. [8] showed a refractory molding composition containing a novel binding agent, which is preferably a co-condensation resin of furfuryl alcohol and organosolv lignin; Liang et al. [9] proposed lignin–furfuryl alcohol resins used as abrasive grinding wheels; Zhang et al. [10] reported that a novel environmentally friendly lignin-based adhesive crosslinked with a furfuryl alcohol–glyoxal resin and epoxy resin was used to bond particleboard. However, there has been little success in their widely industrial production and applications where most attempts are finally toward producing lignin–phenol–formaldehyde (LPF) resin. Its ingredients, phenol and formaldehyde, were environmentally unfriendly and toxic substances, with LD_50_ values in rats of 317 mg/kg and 65 mg/kg, respectively [11]. This lack of success can be attributed to the technical reasons and less basic research. Our structural understanding on these lignin-based furan resins appears less in contrast with the LPF resin.

**Figure 1:**
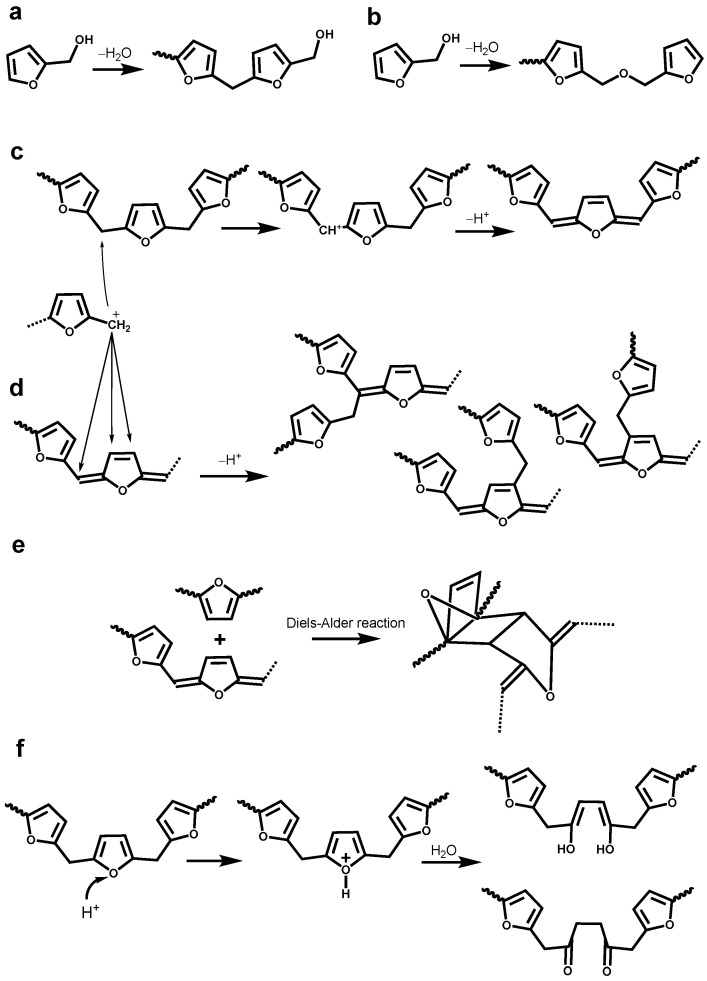
Different pathways for the self-polymerization of furfuryl alcohol: (**a**) formation of methylene linkage; (**b**) formation of dimethyl ether linkage; (**c**) formation of conjugated double-bond sequences; (**d**) formation of branching linkage; (**e**) Diels-Alder cycloaddition reaction; (**f**) formation of γ-diketone structures [18,19,20].

The optimization of LFA resin needs deep understanding of the curing mechanism and structure–property correlations. However, a very complex and extremely heterogeneous structure of technical lignins is resultant from various chemical processes, including kraft cooking [12,13,14,15,16,17], which raises the challenges. ^1^H-^13^C Heteronuclear Single-Quantum Correlation (HSQC) 2D NMR spectrum of KL showed a few hundred signals corresponding to various structural lignin units, such as condensed lignin moieties, products of β-O-4 bond cleavage, vinyl and alkyl-aryl structures, saturated aliphatic moieties, and others [13,14,15,16,17]. In addition, self-polymerization of FA by Brönsted and Lewis acid yields a black crosslinked resin through several complex paths [18]. The first phase of this polymerization consists of condensation reactions between the methylol group from one furan ring and either the position 5 of another furan ring (Figure 1a) or methylol group from another furan ring (Figure 1b) [19,20]. These two pathways only lead to linear oligomeric chains. The formation of branching and crosslinking structures is subordinate to the formation of conjugated double-bond sequences in the linear polymer chains (Figure 1c) and occurs through the interaction of terminal methylol groups or disubstituted furan rings with non-furanic unsturations (Figure 1d,e) [20]. At last, the protonic species can also attack the oxygen atoms of the furan ring resulting the ring opening to form γ-diketone structures (Figure 1f) [19]. Therefore, understanding the reactions between kraft lignin and furfuryl alcohol is obviously a tremendous task but has a practical rational. A comprehensive methodology, including the development of advanced analytical techniques as well as the validation and standardization of new and traditional methods, is critical for the reliable structural characterization of LFA resins.

NMR spectroscopy is widely used in lignin structural characterization nowadays. Advanced qualitative and semiquantitative comprehensive 2D NMR techniques and the express analysis of OH groups by quantitative ^31^P NMR are among efficient analytical methods. The 2D HSQC technique provides a great improvement in signal separation from a very complex lignin polymer, especially those from the side chain. This allows the identification of a large number of structural units, such as β-O-4, β-5, β-β, stilbene, enol ether, and arylglycerol linkages in the HSQC spectra of various lignins [13,14,15,21,22,23]. The HSQC method was also beneficial in the quantification of substructures of lignins [24,25,26]. Therefore, the current methods are well suitable to comparative analyses of lignins and their derivatives.

Wet chemistry methods, such as functional group analysis, are also very useful to determine specific structural moieties in lignins. Methoxyl group is one of the major characteristic functional groups in lignin. The conventional determination of methoxyl groups is based on its acid-catalyzed reaction with iodide ion to form methyl iodide being detected in a variety of ways [27,28]. The methoxyl content could serve as a useful parameter to provide an approximate measure of the lignin content [28,29]. Methoxyl group analysis was used for material balance calculations in this study.

Thus, the objective of this research was to develop a methodology for the structural analysis on LFA bioresin formulated from a softwood KL and FA. The 2D HSQC NMR analysis was used to identify and quantify newly formed CH-moieties in the LFA bioresin. It was supplemented by ^31^P NMR and methoxyl group analysis. Finally, the structural data from above methods suggested that structures formed between lignin and pFA in LFA bioresin.

## 2. Materials and Methods

### 2.1. General

All chemicals and solvents were purchased from Sigma-Aldrich (Espoo, Finland) if not stated otherwise and were used without further purification. Softwood KL was provided from a pulp mill. The naming of lignin structures was based on the traditional numbering system for lignins [30] rather than the systematic IUPAC numbering scheme. The LFA resin was prepared by a reaction of dry KL in FA-water mixture (30:52:18, *w*/*w*, respectively) at 50 °C for 60 min under acidic conditions with stirring at 1000 rpm. Reference pFA was made under the same conditions in the absence of lignin.

### 2.2. Fractionation of LFA Resins

**Figure 2:**
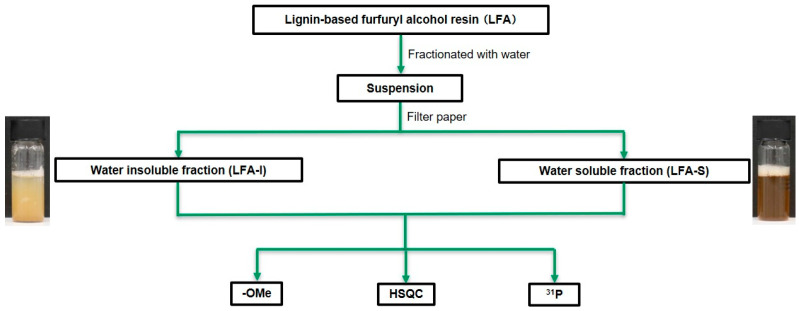
The scheme of fractionation of LFA resin for the structural analysis.

To remove unreacted FA and its side products, water was used to separate the bioresin into water-insoluble (LFA-I) and soluble fractions (LFA-S), assuming FA and its oligomers are soluble in water. More specifically (Figure 2), 100 g of water was slowly added to 3.40 g of the LFA resin with stirring, and the mixture was kept overnight at 25 °C. The suspension was subsequently filtered with a filter paper (No. 4, Kiriyama Glass Works Company Ltd., Tokyo, Japan), resulting in dark-brown-colored LFA-I and LFA-S. LFA-I was dried in a vacuum oven at 25 °C overnight with the aid of P_2_O_5_. LFA-S was rotatory evaporated in vacuo to remove water and FA at 45 °C under 500 rpm (R-210/215, BUCHI Labortechnik AG, Flawil, Switzerland) and oven-dried in vacuum at 25 °C overnight. The yields of the fractions were 29.4% (LFA-I, 1.00 g) and 12.9% (LFA-S, 0.44 g) based on the original LFA.

### 2.3. Determination of Methoxyl Groups

The methoxyl group content was determined according to Goto et al. [28] with some modification. About 30 mg of sample with hydroiodic acid (57% *w*/*w*, 10 mL) and a stirring bar were sealed in a crimp top vial (20 mL) with a rubber septum. The vial was heated in an oil bath at 130 °C for 20 min with stirring. After cooling in an ice bath, propyl iodide (CH_3_CH_2_CH_2_I, 0.05 mmol) in *n*-hexane was added with a syringe through the septum as an internal standard (IS), the sealed vial was opened, and the mixture was extracted with cold *n*-hexane (9 mL). The organic layer was dried over Na_2_SO_4_ and then transferred to a 2.5 mL vial with a Teflon-lined crimp seal cap. The yield of methyl iodide (CH_3_I) was analyzed with a Shimadzu GC-2010 (Shimadzu Company Ltd., Kyoto, Japan) gas chromatograph (GC) equipped with a flame ionization detector (FID) using a standard polysiloxane nonpolar column (HP-5, 30 m × 0.32 mm i.d. × 0.25 µm film thickness; Agilent J&W Scientific, Folsom, CA, USA). The GC analysis was operated under the following conditions: inject volume: 1 µL, carrier gas, helium; split ratio, 50; flow rate, 1.0 mL min^−1^; injection temperature, 200 °C; detector temperature, 230 °C. The oven temperature program was 27 °C for 6 min, heating to 150 °C at 10 °C min^−1^ with the total running time of 18.3 min. The retention time values for CH_3_I and IS were 3.5 min and 7.7 min, respectively. The yield of CH_3_I was calculated using a calibration curve made from authentic CH_3_I and CH_3_CH_2_CH_2_I mixtures in *n*-hexane at different concentrations. The calibration equation was Y = 3.17X + 0.052, where Y was the molar ratio of CH_3_I to the IS, and X is the peak area of CH_3_I to the IS. The methoxyl content was expressed as mass%.

### 2.4. NMR Analysis on Fractionated Bioresin

#### 2.4.1. HSQC NMR

One-bond adiabatic ^1^H—^13^C correlation (HSQC) was performed using a Bruker AVNEO 600 MHz spectrometers (Billerica, MA, USA). About 80 mg of sample was fully dissolved in 0.6 mL of DMSO-d6 according to the method reported in our previous study [12,13]. A sensitivity-enhanced pulse program (hsqcetgpsisp.2) that utilizes shaped pulses for all 180° pulses on proton channel was used in acquisition. First, 1024 data points were acquired from 11 to 0 ppm in F2 (^1^H), with an acquisition time of 77.8 ms, and from 215 to 0 ppm in F1 (^13^C) with 256 increments, 36 scans, and a 2.0 s interscan delay, with a total acquisition time of 5 h 50 min in 298 K. The average value for one-bond *J*-coupling between protons and carbons was set as 145 Hz. Processing the final matrix to 2 k by 1 k data points was performed by QSINE window functions in both F2 and F1. The signals were assigned by referring to the NMR data of lignin model compounds, FA and pFA reported earlier [13,14,15,21,31,32,33,34,35], or predicted with the ChemDraw Professional 19.0 software (PerkinElmer, Waltham, MA, USA) (see Figures S1–S9 in Supplementary Materials). The integrals were normalized to one-third of the integral of the methoxyl (OMe) signals used as an internal reference (see Figures S10–S12 in Supplementary Materials). Therefore, the results were expressed relatively to 100 OMe group. According to the integral value of methoxyl groups (I_OMe_), the amounts of structural units per 100 OMe could be estimated by the following formula,

I_x_% = 3I_x_/I_OMe_ × 100%

where I_x_ is the integral value of corresponding structural units. The integration was made at the same contour level (number of positive contour levels (nlev) = 50, highest contour level (toplev) = 100%, lowest contour level (lev0) = 20).

#### 2.4.2. ^31^P NMR

The ^31^P NMR experiments were performed with a Bruker AVANCE III 400 MHz spectrometers (Billerica, MA, USA) at 298 K [36]. About 40 mg of sample was transferred into a small vial with a screw cap and mixed with 0.4 mL of freshly prepared CDCl_3_-pridine (1:1.6, *v*/*v*) solvent mixture. Subsequently, 0.05 mL of a relaxation reagent, chromium (III) acetylacetonate solution (11.4 mg/mL), and 0.1 mL of IS, endo-N-hydroxyl-5-norbornene-2,3-dicarboximide solution (20 mg/mL) solution in CDCl_3_-pyridine (1:1.6, *v*/*v*) were added. When the sample was completely dissolved in the solvent mixture, 0.1 mL of phosphitylation reagent (2-chloro-4,4,5,5-tertramethyl-1,3,2-dioxaphospholane) was added, and the mixture was vortexed for around 60 s at room temperature to generate phosphorus-tagged product. Finally, 0.1 mL of CDCl_3_ was added to the vial, and the resulting mixture was transferred into an NMR tube. Based on the T1 experiments, a 1.0 s acquisition time and a 5.0 s relaxation delay were used, and 128 scans were collected. The spectra were phased, calibrated, and the baseline was corrected using a linear function. The integral region of ^31^P NMR peaks corresponding to specific OH groups, i.e., aliphatic OH (ca. 151.4–145.1 ppm), 5-substituted guaiacyl (G)-phenolic OH (ca. 145.1–140.4 ppm), unsubstituted G-phenolic OH (ca. 140.4–138.5 ppm), *p*-hydroxyphenyl (H)-phenolic OH (ca. 138.5–136.9 ppm), and COOH (ca. 135.6–133.8 ppm). The quantification of specific OH groups (in mmol/g sample) was done as follows;

X (mmol g ^−1^ sample) = I_x_ × m_IS_/(179.18m_s_ × I_IS_) × 1000

where X is the amount of the specific OH groups; I_X_ and I_IS_ are the integral values of the specific OH groups and the internal standard, respectively; m_s_ and m_IS_ are the masses of the sample and the internal standard; and 179.18 is the molecular weight of the IS (the corresponding chemical shift is 152.0 ppm).

## 3. Results and Discussion

### 3.1. General

Lignin-based furan resin using FA as a crosslinking agent were prepared. For the FA or pFA, which did not react with lignin fragments during preparation, water was used to remove them from LFA. This resulted in the fractionation of LFA into the water-insoluble (LFA-I) and the water-soluble parts (LFA-S) (Figure 2). As KL is not soluble in acidic water, it was expected that KL would be concentrated in LFA-I. However, the below analysis showed that LFA-S also contained a certain amount of KL apparently due to reactions of KL with FA producing water-soluble products.

### 3.2. Material Balance

**Figure 3:**
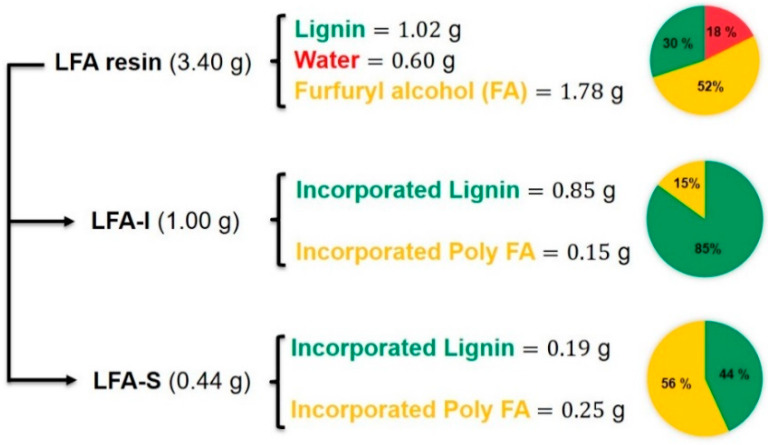
Material balance analysis for LFA resin.

As shown in Figure 3, 1.00 g of dry LFA-I and 0.44 g of dry LFA-S were obtained from 3.40 g of LFA through water fractionation respectively. This LFA was formulated from 30 wt% of dry kraft lignin, 18 wt% of water, and 52 wt% of FA. In order to estimate the lignin content in each fraction, the methoxyl group analysis was used. The methoxyl content of KL was used as the reference.

The methoxyl group is a characteristic functional group of lignin, which could serve as a useful means to provide an approximate measure of lignin content [29]. It was assumed that methoxyl groups did not react during the preparation of bioresin under the mild conditions. Therefore, the lignin amount in each fraction was calculated based on the methoxyl content (Table 1) as 85 wt% and 44 wt% in LFA-I and LFA-S, respectively (Figure 3). The material balance closure was good, indicating that the calculated amount of incorporated kraft lignin in each LFA fractions was reliable. It also showed that about 18% of the original KL ended up in LFA-S and 82% was in LFA-I. In addition, the amount of incorporated pFA was calculated by the difference between the total mass and the amount of lignin as unreacted FA was removed with water during the sample processing. It was only 22% of the amount of FA loaded for the reaction.

### 3.3. Identification of Newly Formed Structures in Bioresin

**Figure 4:**
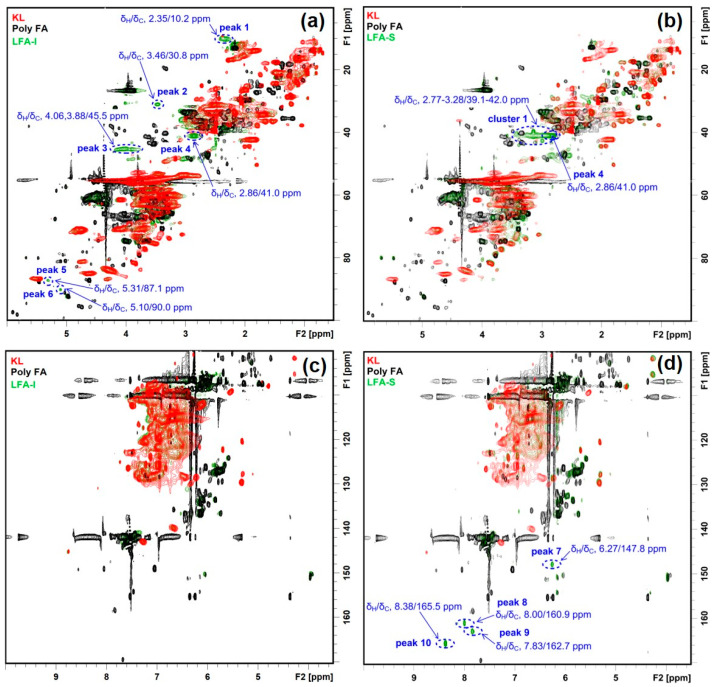
Identified HSQC signals for newly formed structures in LFA fractions, (**a**) aliphatic region of LFA-I fraction, (**b**) aliphatic region of LFA-S fraction, (**c**) aromatic region of LFA-I fraction, (**d**) aromatic region of LFA-S fraction.

The HSQC spectrum of the LFA bioresin was much more complex than the spectrum of lignin–phenol–formaldehyde (LPF) resin reported earlier [37]. In order to recognize signals from newly formed moieties in the LFA bioresin, its HSQC spectra were overlaid with the HSQC spectra of KL and pFA. This showed 10 new peaks and one new cluster in the HSQC spectra of LFA fractions (Figure 4). In detail, peaks 1–6 and cluster 1 were located in the aliphatic region, and peaks 5 and 6 were included in the oxygenated aliphatic region, which seems to be associated with conjugated vinyl moieties. Significant differences in the chemical structure between the LFA fractions were also obvious. Peaks 1–6 were detected in the spectrum of LFA-I, whereas cluster 1 containing peak 4 of high intensities was detected in the spectrum of LFA-S (Figure 4a,b). In addition, peaks 7–10 appeared in the aromatic region of LFA-S but were absent in the spectrum of LFA-I (Figure 4c,d).

### 3.4. Tentative Assignment of New Signals

#### 3.4.1. Aromatic Condensed Structures

**Figure 5:**
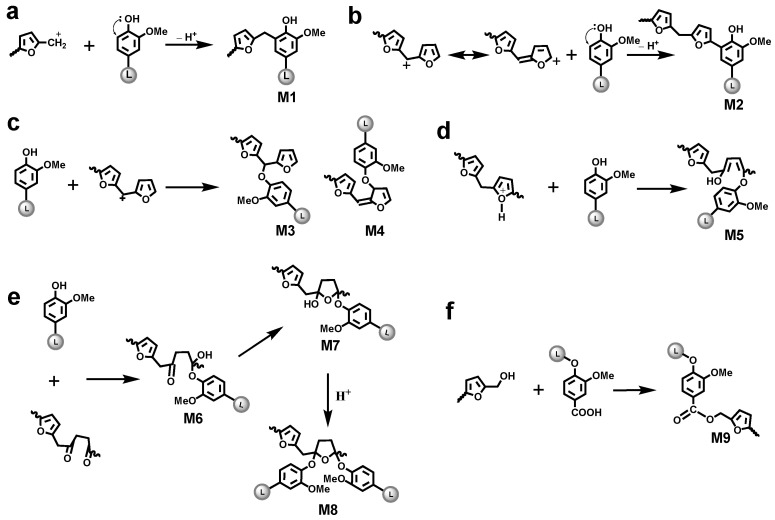
Hypothesized reaction between furfuryl alcohol and lignin (see text for details).

A previous model compound study revealed that a methylene linkage M1 was formed between the 5-position of guaiacyl units and a methylol group of FA/pFA in the presence of acid catalyst (Figure 5a), giving a signal at δ_H_/δ_C_ 3.79/31.7 ppm (in CDCl_3_) [34]. Chemdraw simulation gave a rather similar value (δ_H_/δ_C_ 3.66/29.4 ppm) for this moiety (Figure S1). A similar signal (δ_H_/δ_C_ 3.46/30.8 ppm) was found in the HSQC spectrum of LFA-I (Figure 4a) and therefore assigned to the methylene linkage in M1. However, the signal at δ_H_/δ_C_ 3.46/30.8 ppm was absent in the spectrum of LFA-S.

**Figure 6:**
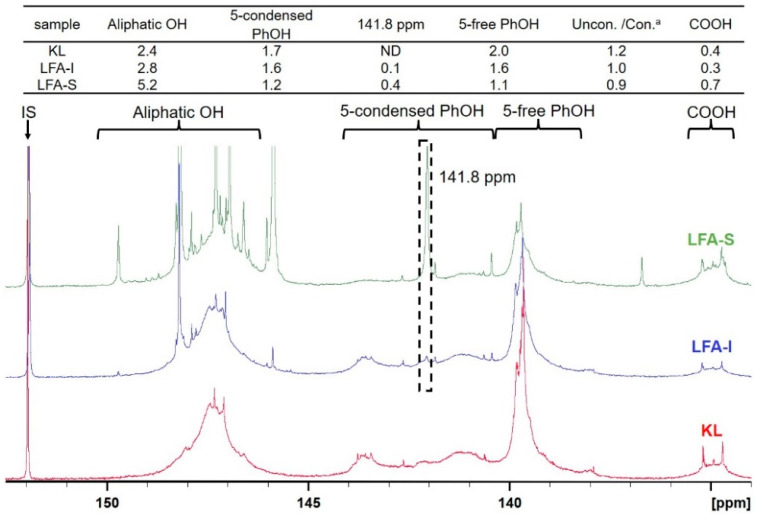
^31^P NMR spectra of the original KL and LFA bioresin fractions and the calculated values (mmol/g). ^a^ Uncon./Con. is the ratio between the amount of 5-free PhOH and 5-condensed PhOH.

Different types of lignin hydroxyl groups as well as carboxylic groups were quantified by ^31^P NMR spectroscopy after phosphitylation (Figure 6). The amounts of aliphatic OH groups dramatically increased due to the contribution of pFA moieties. However, it could be assumed that the resonance in phenolic OH groups belong exclusively to lignin. As the amounts of lignin in LFA fractions were different, it is difficult to compare the amount of PhOH directly. However, the ratio between condensed (at 5-position) and non-condensed PhOH increased in the order KL > LFA-I > LFA-S, indicating participation of the *o*-position (5) in condensation reactions during resin formulation. This was mainly caused by the formation of new condensed phenolic moieties resonating at 141.8 ppm (Figure 6). Their amount was much higher in LFA-S as compared to LFA-I. It could not belong to the structure M1 as it was not detected in the HSQC spectrum of LFA-S. Therefore, the resonance at 141.8 ppm should be originated from other types of 5-condensed structures. For example, a condensed structure M2, where a free phenolic guaiacyl unit is directly connected to a terminal furan ring in pFA, was proposed in Figure 5b. At the first step of this reaction, a carbonium ion located at the 5-position of a terminal furan ring of pFA is formed from an intermediate produced, according to the mechanism shown in Figure 1c through a conjugated double-bond sequence. However, structure M2 does not give any characteristic cross-peak in the HSQC spectrum and therefore cannot be confirmed by this technique.

#### 3.4.2. Aryl Ether Structures

Lignin phenolic hydroxyl oxygen not only donates unshared electrons to the π-system of the aromatic ring to form condensed structures but can also directly participate in the reaction as a nucleophile to produce aryl ether structures with pFA. Herein, several reaction mechanisms between lignin phenolic hydroxyl group and intermediate or moieties of polymering pFA were proposed according to previous reports [19,20]. NMR chemical shifts (CS) of the proposed aryl ether structures were simulated with ChemDraw (Figures S1–S9) and overlaid with the HSQC spectra of the LFA fractions.

*Nucleophilic substitution reaction.* Carbenium ions can be produced at the carbon atoms linking two furan rings or carbon atoms in the furan rings through a successive hydride-ion/proton exchanges (Figure 1c or Figure 5c). The lignin phenolic hydroxyl oxygen with unshared electrons has a possibility to attack these carbenium ions to form aryl ether structures M3 and M4, respectively. In addition, as referred to the formation of γ-diketone structure in Figure 1f, another reaction was proposed in Figure 5d. During acid-catalyzed polymerization, the lignin phenolic hydroxyl oxygen can attack, instead of water molecule, the protonated furan ring with ring opening, forming an aryl ether structure M5.

*Nuclephilic addition reaction.* As discussed above, the γ-diketone structure can be formed during acid catalyzed polymerization of FA. In such case, the phenolic hydroxyl group of lignin can attack the carbonyl groups of this structure to form the hemiacetal or hemiketol (M6). Furthermore, a more stable cyclic hemiacetal or hemiketol (M7) can be transformed from M6, as shown in Figure 5e. At last, these cyclic hemiacetals or hemiketol (M7) can be attacked by the phenolic hydroxyl group to form the acetal or ketol structure (M8).

**Figure 7:**
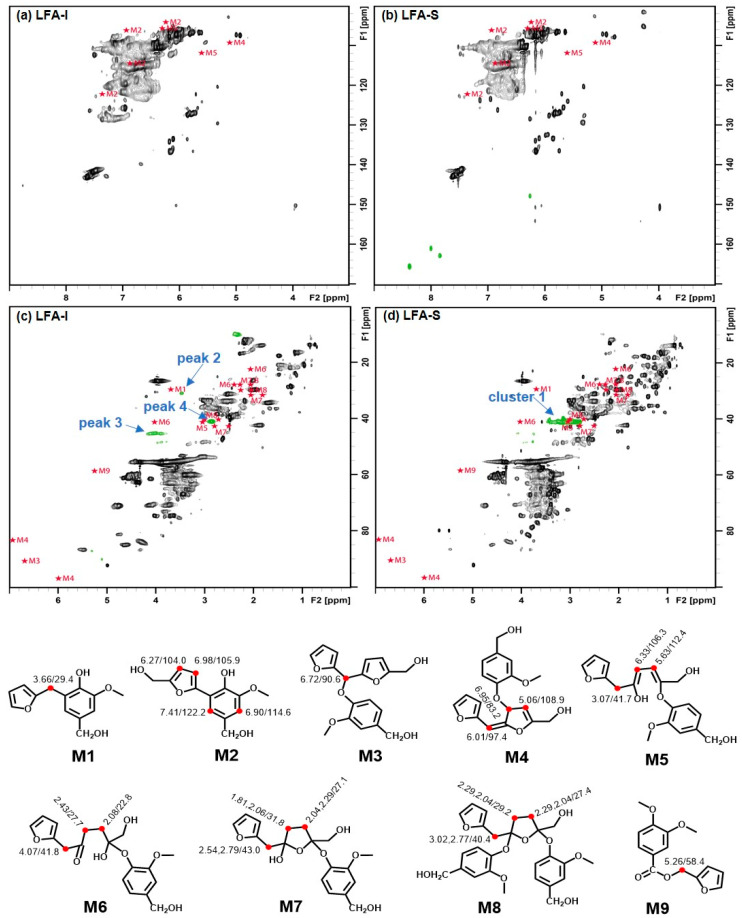
HSQC spectra of LFA fractions overlaid with simulated CS for models M1–9, (**a**) aromatic region of LFA-I, (**b**) aromatic region of LFA-S, (**c**) aliphatic region of LFA-I, (**d**) aliphatic region of LFA-S.

A comparison of the simulated chemical shift (CS) of the proposed aryl ether structures (M3–8) (see Figures S3–S8) with the HSQC spectra of LFA fractions (Figure 7) showed that the calculated CS from models M3–4 were far away from any newly appeared peaks in the LFA resin, whereas the calculated methylene correlation from models M5–8 were close to the peak 3 at δ_H_/δ_C_ 4.06,3.88/45.5 ppm, peak 4 at δ_H_/δ_C_ 2.86/41.0 ppm, and cluster 1 at δ_H_/δ_C_ 2.77–3.28/39.1–42.0 ppm. It indicated a possibility of the formation of various aryl ether structures M5–8 through the nucleophilic addition in LFA resin.

#### 3.4.3. Ester Structure

The carboxylic acid groups are typical for technical lignins. Therefore, under the acidic conditions, they could react with hydroxyl groups in pFA to form ester structures (Figure 5f). This was supported by the ^31^P NMR data showing decreased amounts of COOH groups in LFA-I (Figure 6) (the high amount of COOH in LFA-S was due to the contribution of oxalic acid used as a catalyst in LFA formulation).

### 3.5. Tentative Structure of Bioresin

**Figure 8:**
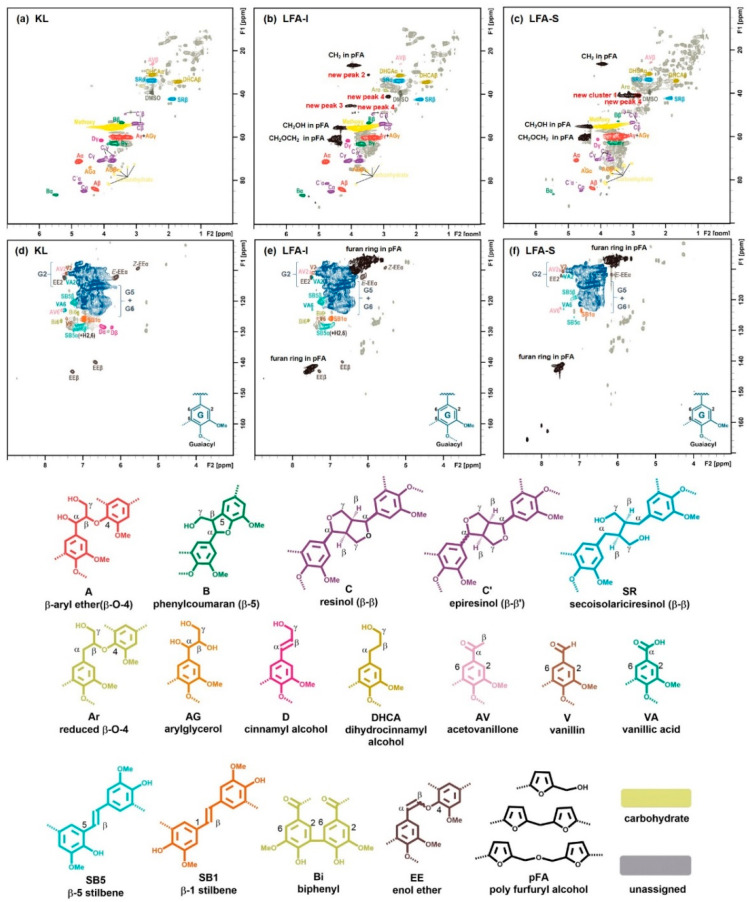
Two-dimensional (2D) HSQC NMR spectra of KL and LFA fractions in DMSO-d6: side-chain region of KL (**a**), LFA-I (**b**) and LFA-S (**c**); aromatic region of KL (**d**), LFA-I (**e**), and LFA-S (**f**), the lev0 is the value of lowest contour level in the spectrum, the colored structures are used to show the main structural linkages in samples (nlev = 50, lev0 = 50, toplev = 100%).

The structural assignment of HSQC spectra of KL and LFA fractions are presented in Figure 8, their main structural quantitative data are shown in Table 2. No significant difference in the oxygenated aliphatic region was observed between KL and LFA. This indicates that the cross-polymerization in the lignin side chain is rather limited and likely occurs in the aromatic ring of lignin, in particular at the 5-position of terminal aromatic rings or/and at the phenolic OH. They are the main reaction types for the cross-polymerization between lignin fragments and pFA chains, finally resulting the LFA resin. Interestingly, the total relative amount of the newly formed structures corresponding cluster 1 is 100 per OMe (Table 2). This can be explained by the high amount of various aryl ether structures corresponding to cluster 1, including an aryl ether structure for peak 4 that may present in the LFA-S fraction. However, the possibility that some structures corresponding cluster 1 are not attached to lignin or/and some other moieties likely contribute to these resonances is not excluded. In contrast, we can observe that a relatively low amount of condensed structure M1, an aryl ether structure corresponding to peaks 3 and 4, are formed in the LFA-I fraction. These results suggest that the degree of crosslinking between pFA and lignin fragments in the LFA-S fraction is higher than that in the LFA-I fraction. The larger amount of pFA in LFA-S quantified with the HSQC method (Table 2) and calculated with material balance (Figure 3), as compared with LFA-I, would support this. This could explain the solubility of the LFA-S fraction in water.

## 4. Conclusions

A methodology for the structural analysis of a lignin-based furan resin with FA (LFA) was developed. The water insoluble and soluble parts of LFA contained 85 wt% and 44 wt% of lignin, respectively. There were significant differences in the structures of these fractions. The HSQC and ^31^P NMR analysis suggested condensation at the 5-position of the aromatic ring; ester formation was also possible. In addition, several aryl ether structures were proposed to be formed based on the comparison of their calculated NMR data with the HSQC spectra of LFA. They are probably the main reaction types for the cross-polymerization between lignin moieties and pFA chains, finally resulting the LFA resin. Therefore, this combination of different analytical methods provided with structural information on bioresin allows product engineering via structure–properties correlation.

## Figures and Tables

**Table 1:** The methoxyl content and calculated lignin content in LFA fractions.

Samples	OMe Content (mmol/g Sample)	Lignin Content (Weight %)
KL	4.12	100
LFA-I	3.52	85
LFA-S	1.84	44

**Table 2:** Tentative assignment of main KL signals and some new signals in the spectra of LFA fractions and their semiquantitative relative quantification (per 100 OMe) (nlev = 50, lev0 = 20, toplev = 100%).

No.	Code	KL	LFA-I	LFA-S	Assignment
1	Methoxyl	100.0	100.0	100.0	—OCH_3_
2	G2	93.0	91.3	91.9	CH-2 in Guaiacyl unit
3	Aα	6.9	6.4	6.0	β-O-4
4	Bα	2.1	2.4	2.0	β-5
5	Cα	1.8	1.9	1.8	β-β
6	C’α	1.3	1.4	1.3	epiresinol and other benzyl-O-Alk ethers
7	SRβ	2.6	2.5	3.2	secoisolariciresinol
8	Arα	0.8	1.0	1.1	reduced β-O-4
9	AGα	1.2	1.1	1.0	arylglycerol
10	Dγ	1.5	1.2	1.2	cinnamyl alcohol
11	DHCAβ	4.1	4.3	4.6	dihydrocinnamyl
12	AVβ	0.3	0.2	0.2	acetovanillone
13	V6	0.9	0.9	0.9	vanillin
14	VA6	0.7	0.5	0.7	vanillic acid
15	Bi6	0.7	1.0	0.8	biphenyl
16	E-EEα	2.4	3.1	3.3	*E*-enol ether
17	Z-EEα	1.0	0.8	nd ^a^	*Z*-enol ether
18	SB1α	3.1	2.8	2.8	β-1 stilbene
19	SB5β	5.3	6.1	5.8	β-5 stilbene
20	CH_2_ in pFA	nd ^a^	37.2	75.4	CH_2_ linkage in pFA
21	CH_2_OH in pFA	nd ^a^	20.8	92.3	terminal CH_2_OH in pFA
22	CH_2_OCH_2_ in pFA	nd ^a^	5.3	12.8	CH_2_OCH_2_ linkage in pFA
23	peak 2	nd ^a^	0.6	nd ^a^	condensed structure
24	peak 3	nd ^a^	2.9	nd ^a^	aryl ether structure
25	peak 4	nd ^a^	2.4	- ^b^	aryl ether structure
26	cluster 1	nd ^a^	nd ^a^	100.1	aryl ether structure

^a^: Not detected; ^b^: included in the cluster 1.

## Data Availability

Data sharing not applicable.

## Supplementary Materials

The following are available online at https://www.mdpi.com/article/10.3390/ma15010350/s1, Figure S1: Calculated NMR spectra of model M1, Figure S2: Calculated NMR spectra of model M2, Figure S3: Calculated NMR spectra of model M3, Figure S4: Calculated NMR spectra of model M4, Figure S5: Calculated NMR spectra of model M5, Figure S6: Calculated NMR spectra of model M6, Figure S7: Calculated NMR spectra of model M7, Figure S8: Calculated NMR spectra of model M8, Figure S9: Calculated NMR spectra of model M9, Figure S10: Integral of typical structures in KL (nlev = 50, lev0 = 20, toplev = 100%), Figure S11: Integral of typical aromatic units in LFA-I (nlev = 50, lev0 = 20, toplev = 100%), Figure S12: Integral of typical aromatic units in LFA-S (nlev = 50, lev0 = 20, toplev = 100%).
