# Supplementary material for: Structural Analysis of Lignin-Based Furan Resin
Source: Materials (Basel). 2022 Jan 4;15(1):350. doi: 10.3390/ma15010350 (PMC8746157; doi:10.3390/ma15010350)
Supplement: Supplementary file 1 [file materials-15-00350-s001.zip › materials-1464209-supplementary.pdf]

Supporting Information (SI) for

Structural Analysis of Lignin-Based Furan Resin

Xuhai Zhu[1*], Bardo Bruijnaers[2], Tainise V. Lourençon[1], Mikhail Balakshin[1*]

[1]Department of Bioproducts and Biosystems, School of Chemical Engineering, Aalto University, Vuorimiehentie 1, Espoo, 02150, Finland

[2]Cellulose-Resin Composite Group, Nemho R&D group, Trespa International B.V., Wetering 20, Weert, 6002 SM, The Netherlands

[*]Mikhail Balakshin – mikhail.balakshin@aalto.fi/Mailing address: PO Box 16300, FI–00076 AALTO, Finland / Phone number: +358–(0)50–308–6570

[*]Xuhai Zhu –E-mail: zhuxh@dicp.ac.cn/Mailing address: Dalian Institute of Chemical Physics, CAS, 457 Zhongshan Road Dalian, China 116023 / Tel.: +86–0411-8437-9846

ChemNMR ^{1}H Estimation

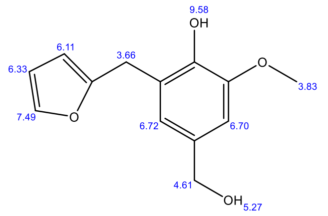

Estimation quality is indicated by color: good, medium, rough

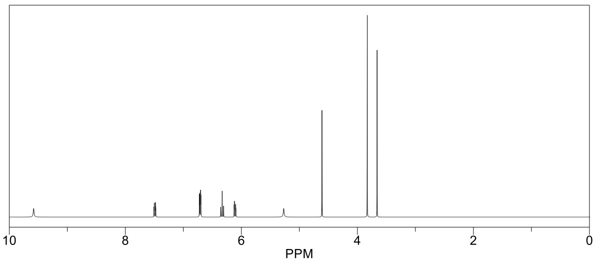


```
Protocol of the H-1 NMR Prediction (Lib=SU Solvent=DMSO 300 MHz):

Node     Shift    Base + Inc.   Comment (ppm rel. to TMS)

OH  9.58              4.20      alcohol
                      4.80        1 -C*R
                      0.58        general corrections
OH  5.27              4.20      alcohol
                      1.10        1 -C-C*R
                     -0.03        general corrections
CH  7.49              7.38      2-furan
                     -0.17        1 -C
                      0.28        general corrections
CH  6.11              6.30      2-furan
                     -0.42        1 -C
                      0.23        general corrections
CH  6.33              6.30      2-furan
                     -0.12        1 -C
                      0.15        general corrections
CH  6.70              7.26      1-benzene
                     -0.38        1 -O-C
                     -0.17        1 -O
                     -0.10        1 -CC*R
                      0.00        1 -C-O
                      0.09        general corrections
CH  6.72              7.26      1-benzene
                     -0.32        1 -O-C
                     -0.17        1 -O
                     -0.10        1 -CC*R
                      0.00        1 -C-O
                      0.05        general corrections
CH2 4.61              1.37      methylene
                      1.22        1 alpha -1:C*C*C*C*C*C*1
                      2.20        1 alpha -O
                     -0.18        general corrections
CH3 3.83              0.86      methyl
                      2.87        1 alpha -O-1:C*C*C*C*C*C*1
                      0.10        general corrections
CH2 3.66              1.37      methylene
                      1.22        1 alpha -1:C*C*C*C*C*C*1
                      1.07        1 alpha -1:C-O-C=C-C=1

1H NMR Coupling Constant Prediction

shift   atom index  coupling partner, constant and vector

9.58        7
5.27       17
7.49       14
                   13   7.5  H-C*C-H
                   12   1.5  H-C*CH*C-H
6.11       12
                   13   7.5  H-C*C-H
                   14   1.5  H-C*CH*C-H
6.33       13
                   14   7.5  H-C*C-H
                   12   7.5  H-C*C-H
6.70        5
                    3   1.5  H-C*C*C-H
6.72        3
                    5   1.5  H-C*C*C-H
4.61       16
3.83        9
3.66       10
```

ChemNMR ^{13}C Estimation

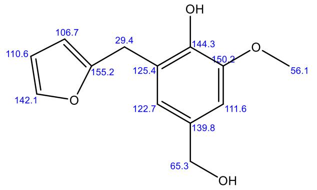

Estimation quality is indicated by color: good, medium, rough

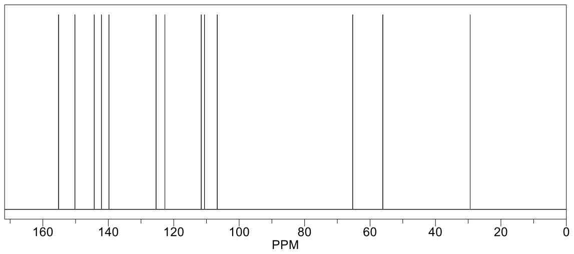


```
Protocol of the C-13 NMR Prediction: (Lib=S)

Node    Shift    Base + Inc.   Comment (ppm rel. to TMS)

C   155.2          143.0      2-furan
                     9.5        1 -C
                     2.7        general corrections
CH  142.1          143.0      2-furan
                    -1.8        1 -C
                     0.9        general corrections
CH  106.7          109.9      2-furan
                    -4.2        1 -C
                     1.0        general corrections
CH  110.6          109.9      2-furan
                     0.7        1 -C
                     0.0        general corrections
C   150.2          128.5      1-benzene
                    33.5        1 -O-C
                   -12.8        1 -O
                    -0.1        1 -C
                     0.2        1 -C-O
                     0.9        general corrections
C   144.3          128.5      1-benzene
                   -14.4        1 -O-C
                    28.8        1 -O
                     0.7        1 -C
                    -1.1        1 -C-O
                     1.8        general corrections
C   125.4          128.5      1-benzene
                     1.0        1 -O-C
                   -12.8        1 -O
                     9.2        1 -C
                     0.2        1 -C-O
                    -0.7        general corrections
C   139.8          128.5      1-benzene
                     1.0        1 -O-C
                    -7.4        1 -O
                    -0.1        1 -C
                    12.4        1 -C-O
                     5.4        general corrections
CH  111.6          128.5      1-benzene
                   -14.4        1 -O-C
                     1.4        1 -O
                    -3.0        1 -C
                    -1.2        1 -C-O
                     0.3        general corrections
CH  122.7          128.5      1-benzene
                    -7.7        1 -O-C
                     1.4        1 -O
                     0.7        1 -C
                    -1.2        1 -C-O
                     1.0        general corrections
CH2 65.3            -2.3      aliphatic
                    24.3        1 alpha -1:C*C*C*C*C*C*1
                    49.0        1 alpha -O
                     0.3        1 delta -C
                     0.3        1 delta -O
                    -6.3        general corrections
CH3 56.1            -2.3      aliphatic
                    49.0        1 alpha -O
                     9.3        1 beta -1:C*C*C*C*C*C*1
                     0.3        1 delta -O
                    -0.2        general corrections
CH2 29.4            -2.3      aliphatic
                    24.3        1 alpha -1:C*C*C*C*C*C*1
                    17.0        1 alpha -1:C-O-C=C-C=1
                    -6.2        1 gamma -O
                     0.3        1 delta -C
                     0.3        1 delta -O
                    -4.0        general corrections
```

Figure S1. calculated NMR spectra of model **M1**

ChemNMR ^{1}H Estimation

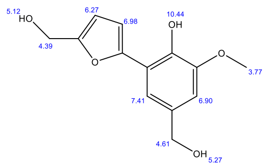

Estimation quality is indicated by color: good, medium, rough

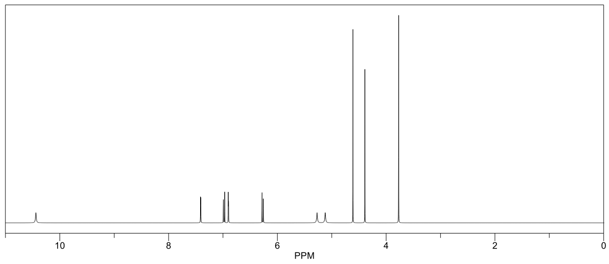


```
Protocol of the H-1 NMR Prediction (Lib=SU Solvent=DMSO 300 MHz):

Node     Shift    Base + Inc.   Comment (ppm rel. to TMS)

OH  10.44            4.20      alcohol
                       4.80       1 -C*R
                       1.44       general corrections
OH   5.12            4.20      alcohol
                       1.10       1 -C-C*R
                      -0.18       general corrections
OH   5.27            4.20      alcohol
                       1.10       1 -C-C*R
                      -0.03       general corrections
CH   6.98            6.30      2-furan
                         ?        1 unknown substituent(s)
                      -0.05       1 -C-O
                       0.73       general corrections
CH   6.27            6.30      2-furan
                         ?        1 unknown substituent(s)
                      -0.11       1 -C-O
                       0.08       general corrections
CH   6.90            7.26      1-benzene
                      -0.38       1 -O-C
                      -0.17       1 -O
                       0.10       1 -C*R
                       0.00       1 -C-O
                       0.09       general corrections
CH   7.41            7.26      1-benzene
                      -0.32       1 -O-C
                      -0.17       1 -O
                       0.34       1 -C*R
                       0.00       1 -C-O
                       0.30       general corrections
CH2 4.39             1.37      methylene
                       1.07       1 alpha -1:C-O-C=C-C=1
                       2.20       1 alpha -O
                      -0.25       general corrections
CH2 4.61             1.37      methylene
                       1.22       1 alpha -1:C*C*C*C*C*C*1
                       2.20       1 alpha -O
                      -0.18       general corrections
CH3 3.77             0.86      methyl
                       2.87       1 alpha -O-1:C*C*C*C*C*C*1
                       0.04       general corrections

1H NMR Coupling Constant Prediction

shift    atom index   coupling partner, constant and vector

10.44        7
5.12        16
5.27        18
6.98        11
                      12    7.5   H-C*C-H
6.27        12
                      11    7.5   H-C*C-H
6.90         5
                       3    1.5   H-C*C*C-H
7.41         3
                       5    1.5   H-C*C*C-H
4.39        15
4.61        17
3.77         9
```

ChemNMR ^{13}C Estimation

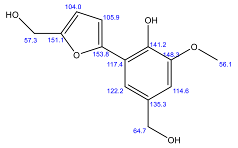

Estimation quality is indicated by color: good, medium, rough

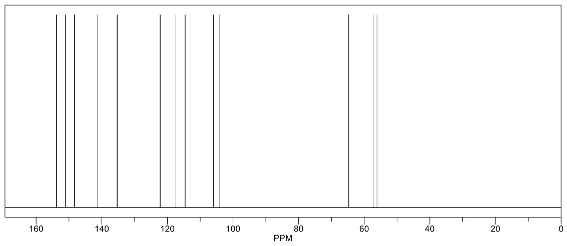


```
Protocol of the C-13 NMR Prediction: (Lib=S)

Node     Shift    Base + Inc.   Comment (ppm rel. to TMS)

C   153.8          143.0      2-furan
                     11.0       1 -1:C*C*C*C*C*C*1
                     -0.2       1 -C-O
                      0.0       general corrections
C   151.1          143.0      2-furan
                     -1.0       1 -1:C*C*C*C*C*C*1
                     11.2       1 -C-O
                     -2.1       general corrections
CH  105.9          109.9      2-furan
                      1.7       1 -1:C*C*C*C*C*C*1
                      0.8       1 -C-O
                     -6.5       general corrections
CH  104.0          109.9      2-furan
                     -4.9       1 -1:C*C*C*C*C*C*1
                     -2.0       1 -C-O
                      1.0       general corrections
C   148.3          128.5      1-benzene
                     33.5       1 -O-C
                    -12.8       1 -O
                      0.5       1 -C*R
                      0.2       1 -C-O
                     -1.6       general corrections
C   141.2          128.5      1-benzene
                    -14.4       1 -O-C
                     28.8       1 -O
                     -1.5       1 -C*R
                     -1.1       1 -C-O
                      0.9       general corrections
C   117.4          128.5      1-benzene
                      1.0       1 -O-C
                    -12.8       1 -O
                      8.0       1 -C*R
                      0.2       1 -C-O
                     -7.5       general corrections
C   135.3          128.5      1-benzene
                      1.0       1 -O-C
                     -7.4       1 -O
                      0.5       1 -C*R
                     12.4       1 -C-O
                      0.3       general corrections
CH  114.6          128.5      1-benzene
                    -14.4       1 -O-C
                      1.4       1 -O
                      0.0       1 -C*R
                     -1.2       1 -C-O
                      0.3       general corrections
CH  122.2          128.5      1-benzene
                     -7.7       1 -O-C
                      1.4       1 -O
                     -1.5       1 -C*R
                     -1.2       1 -C-O
                      2.7       general corrections
CH2 57.3          -2.3      aliphatic
                     17.0       1 alpha -1:C-O-C=C-C=1
                     49.0       1 alpha -O
                      0.3       1 delta -1:C*C*C*C*C*C*1
                     -6.7       general corrections
CH2 64.7          -2.3      aliphatic
                     24.3       1 alpha -1:C*C*C*C*C*C*1
                     49.0       1 alpha -O
                     -0.3       1 delta -1:C-O-C=C-C=1
                      0.3       1 delta -O
                     -6.3       general corrections
CH3 56.1          -2.3      aliphatic
                     49.0       1 alpha -O
                      9.3       1 beta -1:C*C*C*C*C*C*1
                      0.3       1 delta -O
                     -0.2       general corrections
```

Figure S2. calculated NMR spectra of model **M2**

ChemNMR ^{1}H Estimation

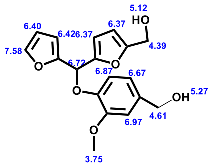

Estimation quality is indicated by color: good, medium, rough

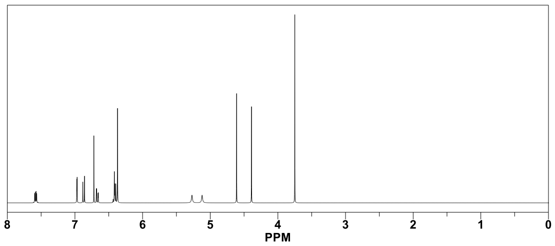


```
Protocol of the H-1 NMR Prediction (Lib=SU Solvent=DMSO 300 MHz):

Node     Shift    Base + Inc.   Comment (ppm rel. to TMS)

OH  5.12               4.20     alcohol
                       1.10       1 -C-C*R
                      -0.18       general corrections
OH  5.27               4.20     alcohol
                       1.10       1 -C-C*R
                      -0.03       general corrections
CH  7.58               7.38     2-furan
                      -0.08       1 -C-O
                       0.28       general corrections
CH  6.37               6.30     2-furan
                      -0.11       1 -C-O
                      -0.05       1 -C-O
                       0.23       general corrections
CH  6.42               6.30     2-furan
                      -0.11       1 -C-O
                       0.23       general corrections
CH  6.37               6.30     2-furan
                      -0.05       1 -C-O
                      -0.11       1 -C-O
                       0.23       general corrections
CH  6.40               6.30     2-furan
                      -0.05       1 -C-O
                       0.15       general corrections
CH  6.97               7.26     1-benzene
                       0.00       1 -O-C
                      -0.38       1 -O-C
                       0.00       1 -C-O
                       0.09       general corrections
CH  6.87               7.26     1-benzene
                      -0.38       1 -O-C
                       0.00       1 -O-C
                       0.03       1 -C-O
                      -0.04       general corrections
CH  6.67               7.26     1-benzene
                       0.00       1 -O-C
                      -0.32       1 -O-C
                       0.00       1 -C-O
                      -0.27       general corrections
CH  6.72               1.50     methine
                       3.02       2 alpha -1:C*O*C*C*C*1
                       2.20       1 alpha -O-1:C*C*C*C*C*C*1
CH2 4.39               1.37     methylene
                       1.07       1 alpha -1:C-O-C=C-C=1
                       2.20       1 alpha -O
                      -0.25       general corrections
CH2 4.61               1.37     methylene
                       1.22       1 alpha -1:C*C*C*C*C*C*1
                       2.20       1 alpha -O
                      -0.18       general corrections
CH3 3.75               0.86     methyl
                       2.87       1 alpha -O-1:C*C*C*C*C*C*1
                       0.02       general corrections

1H NMR Coupling Constant Prediction

shift   atom index  coupling partner, constant and vector

5.12        13
5.27        24
7.58         3
                   2   7.5  H-C*C-H
                   1   1.5  H-C*CH*C-H
6.37        11
                  10   7.5  H-C*C-H
6.42         1
                   2   7.5  H-C*C-H
                   3   1.5  H-C*CH*C-H
6.37        10
                  11   7.5  H-C*C-H
6.40         2
                   3   7.5  H-C*C-H
                   1   7.5  H-C*C-H
6.97        17
                  19   1.5  H-C*C*C-H
6.87        20
                  19   7.5  H-C*C-H
6.67        19
                  20   7.5  H-C*C-H
                  17   1.5  H-C*C*C-H
6.72         6
4.39        12
4.61        23
3.75        22
```

ChemNMR ^{13}C Estimation

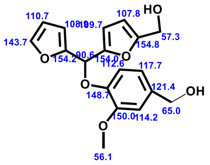

Estimation quality is indicated by color: good, medium, rough

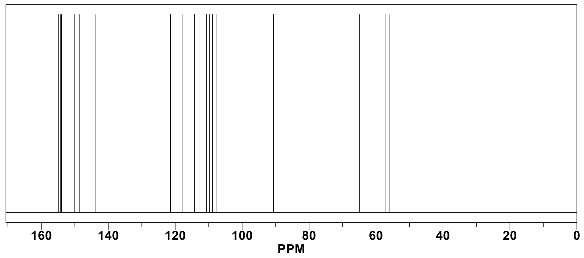


```
Protocol of the C-13 NMR Prediction: (Lib=S)

Node     Shift    Base + Inc.   Comment (ppm rel. to TMS)

C    154.0          143.0     2-furan
                     11.2        1 -C-O
                     -0.2        1 -C-O
C    154.2          143.0     2-furan
                     11.2        1 -C-O
C    154.8          143.0     2-furan
                     -0.2        1 -C-O
                     11.2        1 -C-O
                      0.8        general corrections
CH   143.7          143.0     2-furan
                     -0.2        1 -C-O
                      0.9        general corrections
CH   109.7          109.9     2-furan
                     -2.0        1 -C-O
                      0.8        1 -C-O
                      1.0        general corrections
CH   108.9          109.9     2-furan
                     -2.0        1 -C-O
                      1.0        general corrections
CH   107.8          109.9     2-furan
                      0.8        1 -C-O
                     -2.0        1 -C-O
                     -0.9        general corrections
CH   110.7          109.9     2-furan
                      0.8        1 -C-O
                      0.0        general corrections
C    148.7          128.5     1-benzene
                     33.5        1 -O-C
                    -14.4        1 -O-C
                     -1.1        1 -C-O
                      2.2        general corrections
C    150.0          128.5     1-benzene
                    -14.4        1 -O-C
                     33.5        1 -O-C
                      0.2        1 -C-O
                      2.2        general corrections
C    121.4          128.5     1-benzene
                     -7.7        1 -O-C
                      1.0        1 -O-C
                     12.4        1 -C-O
                    -12.8        general corrections
CH   114.2          128.5     1-benzene
                      1.0        1 -O-C
                    -14.4        1 -O-C
                     -1.2        1 -C-O
                      0.3        general corrections
CH   112.6          128.5     1-benzene
                    -14.4        1 -O-C
                      1.0        1 -O-C
                      0.2        1 -C-O
                     -2.7        general corrections
CH   117.7          128.5     1-benzene
                      1.0        1 -O-C
                     -7.7        1 -O-C
                     -1.2        1 -C-O
                     -2.9        general corrections
CH   90.6            -2.3     aliphatic
                     34.0        2 alpha -1:C-O-C=C-C=1
                     49.0        1 alpha -O
                      9.3        1 beta -1:C*C*C*C*C*C*1
                      0.3        1 delta -C
                      0.3        1 delta -O
CH2  57.3            -2.3     aliphatic
                     17.0        1 alpha -1:C-O-C=C-C=1
                     49.0        1 alpha -O
                      0.3        1 delta -C
                     -6.7        general corrections
CH2  65.0            -2.3     aliphatic
                     24.3        1 alpha -1:C*C*C*C*C*C*1
                     49.0        1 alpha -O
                      0.3        1 delta -O
                     -6.3        general corrections
CH3  56.1            -2.3     aliphatic
                     49.0        1 alpha -O
                      9.3        1 beta -1:C*C*C*C*C*C*1
                      0.3        1 delta -O
                     -0.2        general corrections
```

Figure S3. calculated NMR spectra of model **M3**

ChemNMR ^{1}H Estimation

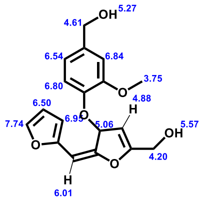

Estimation quality is indicated by color: good, medium, rough

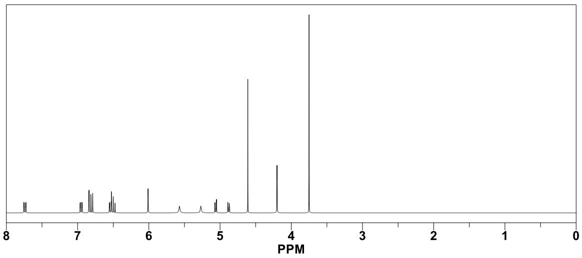


```
Protocol of the H-1 NMR Prediction (Lib=SU Solvent=DMSO 300 MHz):

Node     Shift    Base + Inc.   Comment (ppm rel. to TMS)

OH   5.57              4.20       alcohol
                          0.75       1 -CC=C
                          0.62       general corrections
OH   5.27              4.20       alcohol
                          1.10       1 -C-C*R
                         -0.03       general corrections
CH   7.74              7.38       2-furan
                            ?        1 unknown substituent(s)
                          0.36       general corrections
CH   5.06              1.50       methine
                          1.36       2 alpha -C=C
                          2.20       1 alpha -O-1:C*C*C*C*C*C*1
CH   6.95              6.30       2-furan
                            ?        1 unknown substituent(s)
                          0.65       general corrections
CH   6.50              6.30       2-furan
                            ?        1 unknown substituent(s)
                          0.20       general corrections
CH   6.84              7.26       1-benzene
                         -0.13       1 -O-CC=C
                         -0.38       1 -O-C
                          0.00       1 -C-O
                          0.09       general corrections
CH   6.80              7.26       1-benzene
                         -0.45       1 -O-CC=C
                          0.00       1 -O-C
                          0.03       1 -C-O
                         -0.04       general corrections
CH   6.54              7.26       1-benzene
                         -0.13       1 -O-CC=C
                         -0.32       1 -O-C
                          0.00       1 -C-O
                         -0.27       general corrections
CH2 4.20               1.37       methylene
                          0.63       1 alpha -C=C
                          2.20       1 alpha -O
CH2 4.61               1.37       methylene
                          1.22       1 alpha -1:C*C*C*C*C*C*1
                          2.20       1 alpha -O
                         -0.18       general corrections
CH3 3.75               0.86       methyl
                          2.87       1 alpha -O-1:C*C*C*C*C*C*1
                          0.02       general corrections
H    4.88              5.25       1-ethylene
                         -1.00       1 -O-C=C trans
                         -0.01       1 -C-O cis
                          0.64       1 -C-O gem
H    6.01              5.25       1-ethylene
                         -0.60       1 -O-C=C cis
                         -0.02       1 -C-O trans
                          1.38       1 -C*R gem

1H NMR Coupling Constant Prediction

shift   atom index  coupling partner, constant and vector

5.57       13
5.27       24
7.74        3
                       2    7.5   H-C*C-H
                       1    1.5   H-C*CH*C-H
5.06       11
                      25    6.2   H-C-C(sp2)-H
                      26   -1.0   H-C>C=C>H
6.95        1
                       2    7.5   H-C*C-H
                       3    1.5   H-C*CH*C-H
6.50        2
                       3    7.5   H-C*C-H
                       1    7.5   H-C*C-H
6.84       17
                      19    1.5   H-C*C*C-H
6.80       20
                      19    7.5   H-C*C-H
6.54       19
                      20    7.5   H-C*C-H
                      17    1.5   H-C*C*C-H
4.20       12
                      25   -1.0   H-CH>C=C<H
4.61       23
3.75       22
4.88       25
                      11    6.2   H-C(sp2)-C-H
                      12   -1.0   H>C=C<CH-H
6.01       26
                      11   -1.0   H>C=C>C-H
```

ChemNMR ^{13}C Estimation

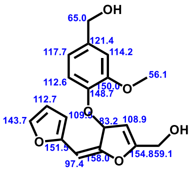

Estimation quality is indicated by color: good, medium, rough

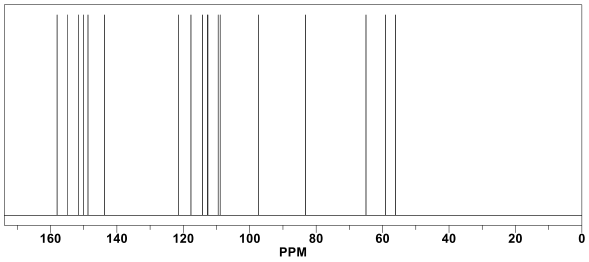


```
Protocol of the C-13 NMR Prediction: (Lib=S)

Node     Shift    Base + Inc.   Comment (ppm rel. to TMS)

C    154.8          123.3       1-ethylene
                       14.2          1 -C-O
                       25.5          1 -OC=C
                       -8.2          1 -C-O
C    151.5          143.0       2-furan
                       12.3          1 -C=C
                       -3.8          general corrections
CH   143.7          143.0       2-furan
                        2.0          1 -C=C
                       -1.3          general corrections
C    158.0          123.3       1-ethylene
                       14.2          1 -C-O
                       25.5          1 -OC=C
                       -5.0          1 -C*R
CH   83.2            -2.3       aliphatic
                       39.0          2 alpha -C=C
                       49.0          1 alpha -O
                        9.3          1 beta -1:C*C*C*C*C*C*1
                       10.1          1 beta -O
                       -5.0          1 gamma -1:C-O-C=C-C=1
                       -2.5          1 gamma -C
                        0.6          2 delta -O
                      -15.0          general corrections
CH   108.9          123.3       1-ethylene
                       -8.2          1 -C-O
                      -30.2          1 -OC=C
                       14.2          1 -C-O
                        9.8          general corrections
CH   109.5          109.9       2-furan
                        1.9          1 -C=C
                       -2.3          general corrections
CH   112.7          109.9       2-furan
                        2.8          1 -C=C
                        0.0          general corrections
C    148.7          128.5       1-benzene
                       33.5          1 -O-C
                      -14.4          1 -O-C
                       -1.1          1 -C-O
                        2.2          general corrections
C    150.0          128.5       1-benzene
                      -14.4          1 -O-C
                       33.5          1 -O-C
                        0.2          1 -C-O
                        2.2          general corrections
C    121.4          128.5       1-benzene
                       -7.7          1 -O-C
                        1.0          1 -O-C
                       12.4          1 -C-O
                      -12.8          general corrections
CH   114.2          128.5       1-benzene
                        1.0          1 -O-C
                      -14.4          1 -O-C
                       -1.2          1 -C-O
                        0.3          general corrections
CH   112.6          128.5       1-benzene
                      -14.4          1 -O-C
                        1.0          1 -O-C
                        0.2          1 -C-O
                       -2.7          general corrections
CH   117.7          128.5       1-benzene
                        1.0          1 -O-C
                       -7.7          1 -O-C
                       -1.2          1 -C-O
                       -2.9          general corrections
CH2 59.1             -2.3       aliphatic
                       19.5          1 alpha -C=C
                       49.0          1 alpha -O
                       10.1          1 beta -O
                       -2.1          1 gamma -C=C
                       -2.5          1 gamma -C
                        0.3          1 delta -O
                      -12.9          general corrections
CH2 65.0             -2.3       aliphatic
                       24.3          1 alpha -1:C*C*C*C*C*C*1
                       49.0          1 alpha -O
                        0.3          1 delta -O
                       -6.3          general corrections
CH3 56.1             -2.3       aliphatic
                       49.0          1 alpha -O
                        9.3          1 beta -1:C*C*C*C*C*C*1
                        0.3          1 delta -O
                       -0.2          general corrections
CH   97.4           123.3       1-ethylene
                       -8.2          1 -C-O
                      -30.2          1 -OC=C
                       12.5          1 -C*R
```

Figure S4. calculated NMR spectra of model **M4**

ChemNMR ^{1}H Estimation

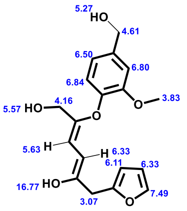

Estimation quality is indicated by color: good, medium, rough

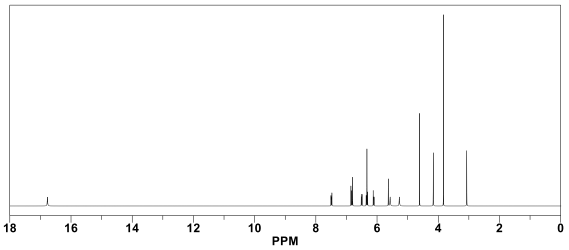


```
Protocol of the H-1 NMR Prediction (Lib=SU Solvent=DMSO 300 MHz):

Node     Shift     Base + Inc.   Comment (ppm rel. to TMS)

OH  5.27              4.20       alcohol
                      1.10          1 -C-C*R
                     -0.03          general corrections
OH  16.77            15.00       enol
                      1.77          general corrections
OH  5.57              4.20       alcohol
                      0.75          1 -CC=C
                      0.62          general corrections
CH  7.49              7.38       2-furan
                     -0.17          1 -C
                      0.28          general corrections
CH  6.11              6.30       2-furan
                     -0.42          1 -C
                      0.23          general corrections
CH  6.33              6.30       2-furan
                     -0.12          1 -C
                      0.15          general corrections
CH  6.80              7.26       1-benzene
                     -0.17          1 -O
                     -0.38          1 -O-C
                      0.00          1 -C-O
                      0.09          general corrections
CH  6.84              7.26       1-benzene
                     -0.53          1 -O
                      0.00          1 -O-C
                      0.03          1 -C-O
                      0.08          general corrections
CH  6.50              7.26       1-benzene
                     -0.17          1 -O
                     -0.32          1 -O-C
                      0.00          1 -C-O
                     -0.27          general corrections
CH2 4.61              1.37       methylene
                      1.22          1 alpha -1:C*C*C*C*C*C*1
                      2.20          1 alpha -O
                     -0.18          general corrections
CH2 4.16              1.37       methylene
                      0.63          1 alpha -C=C
                      2.20          1 alpha -O
                     -0.04          general corrections
CH3 3.83              0.86       methyl
                      2.87          1 alpha -O-1:C*C*C*C*C*C*1
                      0.10          general corrections
CH2 3.07              1.37       methylene
                      1.07          1 alpha -1:C-O-C=C-C=1
                      0.63          1 alpha -C=C
H   5.63              5.25       1-ethylene
                     -0.85          1 -O-1:C*C*C*C*C*C*1 trans
                     -0.01          1 -C-O cis
                      1.24          1 -C=C gem
H   6.33              5.25       1-ethylene
                     -0.22          1 -C cis
                       ?            1 unknown substituent(s)
                      1.24          1 -C=C gem
                      0.06          general corrections

1H NMR Coupling Constant Prediction

shift   atom index   coupling partner, constant and vector

5.27       24
16.77       12
5.57       22
7.49        3
                   2   7.5   H-C*C-H
                   1   1.5   H-C*CH*C-H
6.11        1
                   2   7.5   H-C*C-H
                   3   1.5   H-C*CH*C-H
6.33        2
                   3   7.5   H-C*C-H
                   1   7.5   H-C*C-H
6.80       15
                  17   1.5   H-C*C*C-H
6.84       18
                  17   7.5   H-C*C-H
6.50       17
                  18   7.5   H-C*C-H
                  15   1.5   H-C*C*C-H
4.61       23
4.16       21
                  25  -1.0   H-CH>C=C<H
3.83       20
3.07        6
                  26  -1.0   H-CH>C=C<H
5.63       25
                  21  -1.0   H>C=C<CH-H
6.33       26
                   6  -1.0   H>C=C<CH-H
```

ChemNMR ^{13}C Estimation

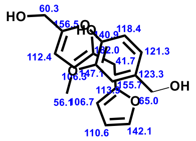

Estimation quality is indicated by color: good, medium, rough

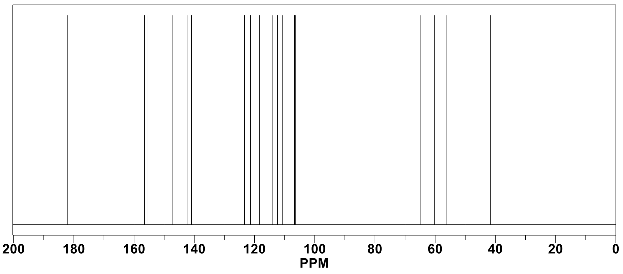


```
Protocol of the C-13 NMR Prediction: (Lib=S)

Node     Shift     Base + Inc.   Comment (ppm rel. to TMS)

C   155.7          143.0       2-furan
                     9.5          1 -C
                     3.2          general corrections
CH  142.1          143.0       2-furan
                    -1.8          1 -C
                     0.9          general corrections
CH  106.7          109.9       2-furan
                    -4.2          1 -C
                     1.0          general corrections
CH  110.6          109.9       2-furan
                     0.7          1 -C
                     0.0          general corrections
C   140.9          128.5       1-benzene
                    28.2          1 -O-C=C
                   -14.4          1 -O-C
                    -1.1          1 -C-O
                    -0.3          general corrections
C   147.1          128.5       1-benzene
                   -11.5          1 -O-C=C
                    33.5          1 -O-C
                     0.2          1 -C-O
                    -3.6          general corrections
C   123.3          128.5       1-benzene
                    -5.8          1 -O-C=C
                     1.0          1 -O-C
                    12.4          1 -C-O
                   -12.8          general corrections
CH  113.9          128.5       1-benzene
                     0.7          1 -O-C=C
                   -14.4          1 -O-C
                    -1.2          1 -C-O
                     0.3          general corrections
CH  118.4          128.5       1-benzene
                   -11.5          1 -O-C=C
                     1.0          1 -O-C
                     0.2          1 -C-O
                     0.2          general corrections
CH  121.3          128.5       1-benzene
                     0.7          1 -O-C=C
                    -7.7          1 -O-C
                    -1.2          1 -C-O
                     1.0          general corrections
CH2 65.0            -2.3       aliphatic
                    24.3          1 alpha -1:C*C*C*C*C*C*1
                    49.0          1 alpha -O
                     0.3          1 delta -O
                    -6.3          general corrections
C   156.5          123.3       1-ethylene
                    14.2          1 -C-O
                    26.0          1 -O-1:C*C*C*C*C*C*1
                    -7.0          1 -C=C
C   182.0          123.3       1-ethylene
                     9.4          1 -C
                    52.7          1 -O
                    -7.0          1 -C=C
                     3.6          general corrections
CH2 60.3            -2.3       aliphatic
                    19.5          1 alpha -C=C
                    49.0          1 alpha -O
                    10.1          1 beta -O
                    -2.6          1 gamma -1:C*C*C*C*C*C*1
                    -2.1          1 gamma -C=C
                   -11.3          general corrections
CH3 56.1            -2.3       aliphatic
                    49.0          1 alpha -O
                     9.3          1 beta -1:C*C*C*C*C*C*1
                     0.3          1 delta -O
                    -0.2          general corrections
CH2 41.7            -2.3       aliphatic
                    17.0          1 alpha -1:C-O-C=C-C=1
                    19.5          1 alpha -C=C
                    10.1          1 beta -O
                    -2.1          1 gamma -C=C
                    -0.5          general corrections
CH  112.4          123.3       1-ethylene
                    -8.2          1 -C-O
                   -28.4          1 -O-1:C*C*C*C*C*C*1
                    13.6          1 -C=C
                    12.1          general corrections
CH  106.3          123.3       1-ethylene
                    -7.4          1 -C
                   -35.3          1 -O
                    13.6          1 -C=C
                    12.1          general corrections
```

Figure S5. calculated NMR spectra of model **M5**

ChemNMR ^{1}H Estimation

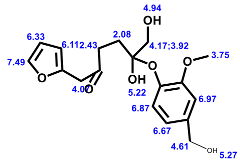

Estimation quality is indicated by color: good, medium, rough

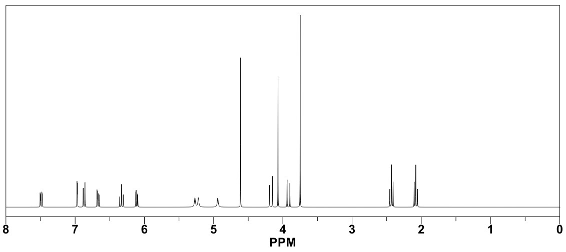


```
Protocol of the H-1 NMR Prediction (Lib=SU Solvent=DMSO 300 MHz):


Node     Shift    Base + Inc.   Comment (ppm rel. to TMS)

OH  5.22              4.20      alcohol
                        1.20       1 -CCO
                       -0.18       general corrections
OH  5.27              4.20      alcohol
                        1.10       1 -C-C*R
                       -0.03       general corrections
OH  4.94              4.20      alcohol
                        1.20       1 -CCO
                       -0.46       general corrections
CH  7.49              7.38      2-furan
                       -0.17       1 -C
                        0.28       general corrections
CH  6.11              6.30      2-furan
                       -0.42       1 -C
                        0.23       general corrections
CH  6.33              6.30      2-furan
                       -0.12       1 -C
                        0.15       general corrections
CH  6.97              7.26      1-benzene
                        0.00       1 -O-C
                       -0.38       1 -O-C
                        0.00       1 -C-O
                        0.09       general corrections
CH  6.87              7.26      1-benzene
                       -0.38       1 -O-C
                        0.00       1 -O-C
                        0.03       1 -C-O
                       -0.04       general corrections
CH  6.67              7.26      1-benzene
                        0.00       1 -O-C
                       -0.32       1 -O-C
                        0.00       1 -C-O
                       -0.27       general corrections
CH2 4.61              1.37      methylene
                        1.22       1 alpha -1:C*C*C*C*C*C*1
                        2.20       1 alpha -O
                       -0.18       general corrections
CH2 4.17,3.915000       1.37       methylene
                        2.20       1 alpha -O
                        0.38       1 beta -O-1:C*C*C*C*C*C*1
                        0.15       1 beta -O
                       -0.06       1 beta -C
CH3 3.75              0.86      methyl
                        2.87       1 alpha -O-1:C*C*C*C*C*C*1
                        0.02       general corrections
CH2 4.07              1.37      methylene
                        1.07       1 alpha -1:C-O-C=C-C=1
                        1.12       1 alpha -C(=O)-C
                        0.51       general corrections
CH2 2.43              1.37      methylene
                        1.12       1 alpha -C(=O)-C
                       -0.06       1 beta -C
CH2 2.08              1.37      methylene
                        0.38       1 beta -O-1:C*C*C*C*C*C*1
                        0.15       1 beta -O
                       -0.06       1 beta -C
                        0.24       1 beta -C(=O)-C

1H NMR Coupling Constant Prediction

shift   atom index  coupling partner, constant and vector

5.22        8
5.27       25
4.94       23
7.49        3
                  2   7.5  H-C*C-H
                  1   1.5  H-C*CH*C-H
6.11        1
                  2   7.5  H-C*C-H
                  3   1.5  H-C*CH*C-H
6.33        2
                  3   7.5  H-C*C-H
                  1   7.5  H-C*C-H
6.97       18
                 16   1.5  H-C*C*C-H
6.87       15
                 16   7.5  H-C*C-H
6.67       16
                 15   7.5  H-C*C-H
                 18   1.5  H-C*C*C-H
4.61       24
4.04       22 diastereotopic -12.4  H-C-H
3.75       21
4.07        6
2.43       11
                 10   7.1  H-CH-CH-H
2.08       10
                 11   7.1  H-CH-CH-H
```

ChemNMR ^{13}C Estimation

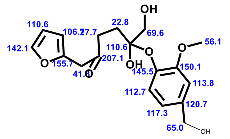

Estimation quality is indicated by color: good, medium, rough

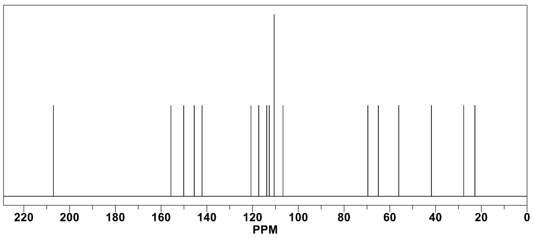


```
Protocol of the C-13 NMR Prediction: (Lib=S)


Node     Shift    Base + Inc.   Comment (ppm rel. to TMS)

C   155.7            143.0      2-furan
                        9.5        1 -C
                        3.2        general corrections
CH  142.1            143.0      2-furan
                       -1.8        1 -C
                        0.9        general corrections
CH  106.7            109.9      2-furan
                       -4.2        1 -C
                        1.0        general corrections
CH  110.6            109.9      2-furan
                        0.7        1 -C
                        0.0        general corrections
C   145.5            128.5      1-benzene
                       30.3        1 -OCC
                      -14.4        1 -O-C
                       -1.1        1 -C-O
                        2.2        general corrections
C   150.1            128.5      1-benzene
                      -14.3        1 -OCC
                       33.5        1 -O-C
                        0.2        1 -C-O
                        2.2        general corrections
C   120.7            128.5      1-benzene
                       -8.4        1 -OCC
                        1.0        1 -O-C
                       12.4        1 -C-O
                      -12.8        general corrections
CH  113.8            128.5      1-benzene
                        0.6        1 -OCC
                      -14.4        1 -O-C
                       -1.2        1 -C-O
                        0.3        general corrections
CH  112.7            128.5      1-benzene
                      -14.3        1 -OCC
                        1.0        1 -O-C
                        0.2        1 -C-O
                       -2.7        general corrections
CH  117.3            128.5      1-benzene
                        0.6        1 -OCC
                       -7.7        1 -O-C
                       -1.2        1 -C-O
                       -2.9        general corrections
C   207.1            193.0      1-carbonyl
                        7.6        1 -C-C
                        6.5        1 -C
                        0.0        general corrections
C   110.6             -2.3      aliphatic
                       18.2        2 alpha -C
                       98.0        2 alpha -O
                        9.3        1 beta -1:C*C*C*C*C*C*1
                        9.4        1 beta -C
                       10.1        1 beta -O
                       -2.7        1 gamma -C(=O)-C
                        0.3        1 delta -O
                      -29.7        general corrections
CH2 65.0              -2.3      aliphatic
                       24.3        1 alpha -1:C*C*C*C*C*C*1
                       49.0        1 alpha -O
                        0.3        1 delta -O
                       -6.3        general corrections
CH2 69.6              -2.3      aliphatic
                        9.1        1 alpha -C
                       49.0        1 alpha -O
                        9.4        1 beta -C
                       20.2        2 beta -O
                       -2.6        1 gamma -1:C*C*C*C*C*C*1
                       -2.5        1 gamma -C
                        0.0        1 delta -C(=O)-C
                      -10.7        general corrections
CH3 56.1              -2.3      aliphatic
                       49.0        1 alpha -O
                        9.3        1 beta -1:C*C*C*C*C*C*1
                        0.3        1 delta -O
                       -0.2        general corrections
CH2 41.8              -2.3      aliphatic
                       17.0        1 alpha -1:C-O-C=C-C=1
                       29.3        1 alpha -C(=O)-C
                       -2.5        1 gamma -C
                        0.3        1 delta -C
CH2 27.7              -2.3      aliphatic
                       29.3        1 alpha -C(=O)-C
                        9.1        1 alpha -C
                        9.4        1 beta -C
                       -5.0        1 gamma -1:C-O-C=C-C=1
                       -2.5        1 gamma -C
                      -12.4        2 gamma -O
                        0.3        1 delta -1:C*C*C*C*C*C*1
                        0.3        1 delta -O
                        1.5        general corrections
CH2 22.8              -2.3      aliphatic
                       18.2        2 alpha -C
                        0.5        1 beta -C(=O)-C
                        9.4        1 beta -C
                       20.2        2 beta -O
                       -2.6        1 gamma -1:C*C*C*C*C*C*1
                       -6.2        1 gamma -O
                       -0.3        1 delta -1:C-O-C=C-C=1
                      -14.1        general corrections
```

Figure S6. calculated NMR spectra of model **M6**

ChemNMR ^{1}H Estimation

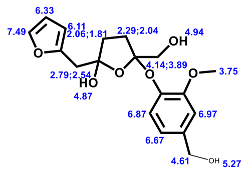

Estimation quality is indicated by color: good, medium, rough

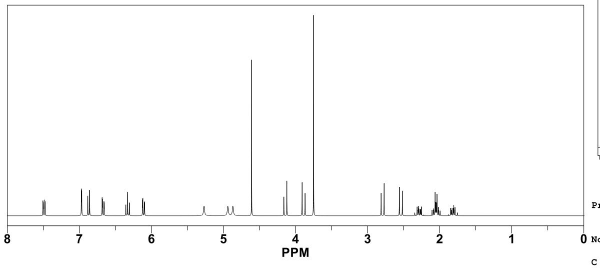


```
Protocol of the H-1 NMR Prediction (Lib=SU Solvent=DMSO 300 MHz):


Node     Shift    Base + Inc.   Comment (ppm rel. to TMS)

OH  4.87              4.20      alcohol
                       0.85        1 -CCC*R
                      -0.18        general corrections
OH  4.94              4.20      alcohol
                       1.20        1 -CCO
                      -0.46        general corrections
OH  5.27              4.20      alcohol
                       1.10        1 -C-C*R
                      -0.03        general corrections
CH  7.49              7.38      2-furan
                      -0.17        1 -C
                       0.28        general corrections
CH  6.11              6.30      2-furan
                      -0.42        1 -C
                       0.23        general corrections
CH  6.33              6.30      2-furan
                      -0.12        1 -C
                       0.15        general corrections
CH2 2.29,2.045000      1.85        tetrahydrofuran
                       0.38        1 beta -O-1:C*C*C*C*C*C*1 from methylene
                      -0.06        1 beta -C from methylene
CH2 2.06,1.815000      1.85        tetrahydrofuran
                       0.15        1 beta -O from methylene
                      -0.06        1 beta -C from methylene
CH  6.97              7.26      1-benzene
                       0.00        1 -O-C
                      -0.38        1 -O-C
                       0.00        1 -C-O
                       0.09        general corrections
CH  6.87              7.26      1-benzene
                      -0.38        1 -O-C
                       0.00        1 -O-C
                       0.03        1 -C-O
                      -0.04        general corrections
CH  6.67              7.26      1-benzene
                       0.00        1 -O-C
                      -0.32        1 -O-C
                       0.00        1 -C-O
                      -0.27        general corrections
CH2 4.14,3.895000      1.37        methylene
                       2.20        1 alpha -O
                       0.38        1 beta -O-1:C*C*C*C*C*C*1
                       0.13        1 beta -O-C
                      -0.06        1 beta -C
CH2 4.61              1.37      methylene
                       1.22        1 alpha -1:C*C*C*C*C*C*1
                       2.20        1 alpha -O
                      -0.18        general corrections
CH3 3.75              0.86      methyl
                       2.87        1 alpha -O-1:C*C*C*C*C*C*1
                       0.02        general corrections
CH2 2.79,2.535000      1.37        methylene
                       1.07        1 alpha -1:C-O-C=C-C=1
                       0.13        1 beta -O-C
                       0.15        1 beta -O
                      -0.06        1 beta -C
```

```
1H NMR Coupling Constant Prediction

shift   atom index  coupling partner, constant and vector

4.87       11
4.94       23
5.27       25
7.49        3
                  2   7.5  H-C*C-H
                  1   1.5  H-C*CH*C-H
6.11        1
                  2   7.5  H-C*C-H
                  3   1.5  H-C*CH*C-H
6.33        2
                  3   7.5  H-C*C-H
                  1   7.5  H-C*C-H
2.17        9 diastereotopic -12.4  H-C-H
                 10   7.1  H-CH-CH-H
1.94       10 diastereotopic -12.4  H-C-H
                  9   7.1  H-CH-CH-H
6.97       18
                 16   1.5  H-C*C*C-H
6.87       15
                 16   7.5  H-C*C-H
6.67       16
                 15   7.5  H-C*C-H
                 18   1.5  H-C*C*C-H
4.02       22 diastereotopic -12.4  H-C-H
4.61       24
3.75       21
2.66        6 diastereotopic -12.4  H-C-H
```

ChemNMR ^{13}C Estimation

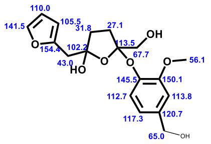

Estimation quality is indicated by color: good, medium, rough

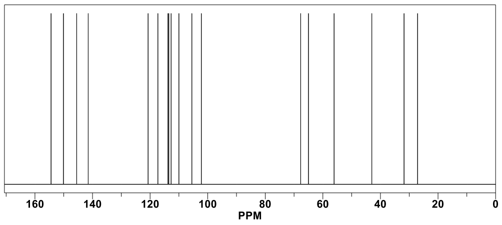


```
Protocol of the C-13 NMR Prediction: (Lib=S)


Node     Shift    Base + Inc.   Comment (ppm rel. to TMS)

C   113.5           -8.5      tetrahydrofuran
                     18.2        2 alpha -C from aliphatic
                     98.0        2 alpha -O from aliphatic
                      9.3        1 beta -1:C*C*C*C*C*C*1 from aliphatic
                     18.8        2 beta -C from aliphatic
                     10.1        1 beta -O from aliphatic
                     -2.5        1 gamma -C from aliphatic
                     -6.2        1 gamma -O from aliphatic
                     -0.3        1 delta -1:C-O-C=C-C=1 from aliphatic
                      0.3        1 delta -O from aliphatic
                    -23.7        general corrections
C   102.2           -8.5      tetrahydrofuran
                     18.2        2 alpha -C from aliphatic
                     98.0        2 alpha -O from aliphatic
                      7.5        1 beta -1:C-O-C=C-C=1 from aliphatic
                     18.8        2 beta -C from aliphatic
                     -2.5        1 gamma -C from aliphatic
                     -6.2        1 gamma -O from aliphatic
                      0.3        1 delta -1:C*C*C*C*C*C*1 from aliphatic
                      0.3        1 delta -O from aliphatic
                    -23.7        general corrections
C   154.4          143.0      2-furan
                     13.6        1 -CCCCC
                     -2.2        general corrections
CH  141.5          143.0      2-furan
                     -2.4        1 -CCCCC
                      0.9        general corrections
CH  105.5          109.9      2-furan
                     -5.4        1 -CCCCC
                      1.0        general corrections
CH  110.0          109.9      2-furan
                      0.1        1 -CCCCC
                      0.0        general corrections
CH2 27.1           -11.2      tetrahydrofuran
                     18.2        2 alpha -C from aliphatic
                     18.8        2 beta -C from aliphatic
                     20.2        2 beta -O from aliphatic
                     -2.6        1 gamma -1:C*C*C*C*C*C*1 from aliphatic
                     -2.5        1 gamma -C from aliphatic
                    -12.4        2 gamma -O from aliphatic
                     -0.3        1 delta -1:C-O-C=C-C=1 from aliphatic
                     -1.1        general corrections
CH2 31.8           -11.2      tetrahydrofuran
                     18.2        2 alpha -C from aliphatic
                     18.8        2 beta -C from aliphatic
                     20.2        2 beta -O from aliphatic
                     -5.0        1 gamma -1:C-O-C=C-C=1 from aliphatic
                     -2.5        1 gamma -C from aliphatic
                     -6.2        1 gamma -O from aliphatic
                      0.3        1 delta -1:C*C*C*C*C*C*1 from aliphatic
                      0.3        1 delta -O from aliphatic
                     -1.1        general corrections
C   145.5          128.5      1-benzene
                     30.3        1 -OCC
                    -14.4        1 -O-C
                     -1.1        1 -C-O
                      2.2        general corrections
C   150.1          128.5      1-benzene
                    -14.3        1 -OCC
                     33.5        1 -O-C
                      0.2        1 -C-O
                      2.2        general corrections
C   120.7          128.5      1-benzene
                     -8.4        1 -OCC
                      1.0        1 -O-C
                     12.4        1 -C-O
                    -12.8        general corrections
CH  113.8          128.5      1-benzene
                      0.6        1 -OCC
                    -14.4        1 -O-C
                     -1.2        1 -C-O
                      0.3        general corrections
CH  112.7          128.5      1-benzene
                    -14.3        1 -OCC
                      1.0        1 -O-C
                      0.2        1 -C-O
                     -2.7        general corrections
CH  117.3          128.5      1-benzene
                      0.6        1 -OCC
                     -7.7        1 -O-C
                     -1.2        1 -C-O
                     -2.9        general corrections
CH2 67.7            -2.3      aliphatic
                      9.1        1 alpha -C
                     49.0        1 alpha -O
                      9.4        1 beta -C
                     20.2        2 beta -O
                     -2.6        1 gamma -1:C*C*C*C*C*C*1
                     -5.0        2 gamma -C
                      0.3        1 delta -C
                      0.3        1 delta -O
                    -10.7        general corrections
CH2 65.0            -2.3      aliphatic
                     24.3        1 alpha -1:C*C*C*C*C*C*1
                     49.0        1 alpha -O
                      0.3        1 delta -O
                     -6.3        general corrections
CH3 56.1            -2.3      aliphatic
                     49.0        1 alpha -O
                      9.3        1 beta -1:C*C*C*C*C*C*1
                      0.3        1 delta -O
                     -0.2        general corrections
CH2 43.0            -2.3      aliphatic
                     17.0        1 alpha -1:C-O-C=C-C=1
                      9.1        1 alpha -C
                      9.4        1 beta -C
                     20.2        2 beta -O
                     -5.0        2 gamma -C
                      0.3        1 delta -C
                      0.3        1 delta -O
                     -6.0        general corrections
```

Figure S7. calculated NMR spectra of model **M7**

ChemNMR ^{1}H Estimation

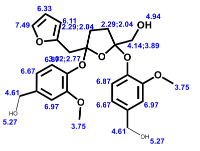

Estimation quality is indicated by color: good, medium, rough

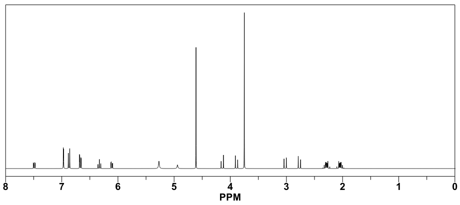


```
Protocol of the H-1 NMR Prediction (Lib=SU Solvent=DMSO 300 MHz):

Node      Shift    Base + Inc.   Comment (ppm rel. to TMS)

OH  4.94              4.20       alcohol
                        1.20        1 -CCO
                       -0.46        general corrections
OH  5.27              4.20       alcohol
                        1.10        1 -C-C*R
                       -0.03        general corrections
OH  5.27              4.20       alcohol
                        1.10        1 -C-C*R
                       -0.03        general corrections
CH  7.49              7.38       2-furan
                       -0.17        1 -C
                        0.28        general corrections
CH  6.11              6.30       2-furan
                       -0.42        1 -C
                        0.23        general corrections
CH  6.33              6.30       2-furan
                       -0.12        1 -C
                        0.15        general corrections
CH2 2.29,2.045000       1.85        tetrahydrofuran
                        0.38        1 beta -O-1:C*C*C*C*C*C*1 from methylene
                       -0.06        1 beta -C from methylene
CH2 2.29,2.045000       1.85        tetrahydrofuran
                        0.38        1 beta -O-1:C*C*C*C*C*C*1 from methylene
                       -0.06        1 beta -C from methylene
CH  6.97              7.26       1-benzene
                        0.00        1 -O-C
                       -0.38        1 -O-C
                        0.00        1 -C-O
                        0.09        general corrections
CH  6.87              7.26       1-benzene
                       -0.38        1 -O-C
                        0.00        1 -O-C
                        0.03        1 -C-O
                       -0.04        general corrections
CH  6.97              7.26       1-benzene
                        0.00        1 -O-C
                       -0.38        1 -O-C
                        0.00        1 -C-O
                        0.09        general corrections
CH  6.67              7.26       1-benzene
                        0.00        1 -O-C
                       -0.32        1 -O-C
                        0.00        1 -C-O
                       -0.27        general corrections
CH  6.87              7.26       1-benzene
                       -0.38        1 -O-C
                        0.00        1 -O-C
                        0.03        1 -C-O
                       -0.04        general corrections
CH  6.67              7.26       1-benzene
                        0.00        1 -O-C
                       -0.32        1 -O-C
                        0.00        1 -C-O
                       -0.27        general corrections
CH2 4.14,3.895000       1.37        methylene
                        2.20        1 alpha -O
                        0.38        1 beta -O-1:C*C*C*C*C*C*1
                        0.13        1 beta -O-C
                       -0.06        1 beta -C
CH2 4.61              1.37       methylene
                        1.22        1 alpha -1:C*C*C*C*C*C*1
                        2.20        1 alpha -O
                       -0.18        general corrections
CH2 4.61              1.37       methylene
                        1.22        1 alpha -1:C*C*C*C*C*C*1
                        2.20        1 alpha -O
                       -0.18        general corrections
CH3 3.75              0.86       methyl
                        2.87        1 alpha -O-1:C*C*C*C*C*C*1
                        0.02        general corrections
CH3 3.75              0.86       methyl
                        2.87        1 alpha -O-1:C*C*C*C*C*C*1
                        0.02        general corrections
CH2 3.02,2.765000       1.37        methylene
                        1.07        1 alpha -1:C-O-C=C-C=1
                        0.38        1 beta -O-1:C*C*C*C*C*C*1
                        0.13        1 beta -O-C
                       -0.06        1 beta -C

1H NMR Coupling Constant Prediction

shift   atom index  coupling partner, constant and vector

4.94        31
5.27        33
5.27        35
7.49         3
                    2   7.5  H-C*C-H
                    1   1.5  H-C*CH*C-H
6.11         1
                    2   7.5  H-C*C-H
                    3   1.5  H-C*CH*C-H
6.33         2
                    3   7.5  H-C*C-H
                    1   7.5  H-C*C-H
2.17         9 diastereotopic -12.4  H-C-H
                   10   7.1  H-CH-CH-H
2.17        10 diastereotopic -12.4  H-C-H
                    9   7.1  H-CH-CH-H
6.97        25
                   23   1.5  H-C*C*C-H
6.87        22
                   23   7.5  H-C*C-H
6.97        16
                   14   1.5  H-C*C*C-H
6.67        23
                   22   7.5  H-C*C-H
                   25   1.5  H-C*C*C-H
6.87        13
                   14   7.5  H-C*C-H
6.67        14
                   13   7.5  H-C*C-H
                   16   1.5  H-C*C*C-H
4.02        30 diastereotopic -12.4  H-C-H
4.61        32
4.61        34
3.75        19
3.75        28
2.89         6 diastereotopic -12.4  H-C-H
```

ChemNMR ^{13}C Estimation

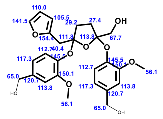

Estimation quality is indicated by color: good, medium, rough

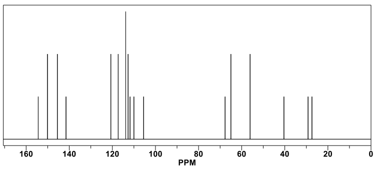


```
Protocol of the C-13 NMR Prediction: (Lib=S)

Node     Shift    Base + Inc.   Comment (ppm rel. to TMS)

C   113.8           -8.5      tetrahydrofuran
                      18.2        2 alpha -C from aliphatic
                      98.0        2 alpha -O from aliphatic
                       9.3        1 beta -1:C*C*C*C*C*C*1 from aliphatic
                      18.8        2 beta -C from aliphatic
                      10.1        1 beta -O from aliphatic
                      -2.5        1 gamma -C from aliphatic
                      -6.2        1 gamma -O from aliphatic
                       0.3        1 delta -1:C*C*C*C*C*C*1 from aliphatic
                      -0.3        1 delta -1:C-O-C=C-C=1 from aliphatic
                       0.3        1 delta -O from aliphatic
                     -23.7        general corrections
C   111.8           -8.5      tetrahydrofuran
                      18.2        2 alpha -C from aliphatic
                      98.0        2 alpha -O from aliphatic
                       9.3        1 beta -1:C*C*C*C*C*C*1 from aliphatic
                       7.5        1 beta -1:C-O-C=C-C=1 from aliphatic
                      18.8        2 beta -C from aliphatic
                      -2.5        1 gamma -C from aliphatic
                      -6.2        1 gamma -O from aliphatic
                       0.3        1 delta -1:C*C*C*C*C*C*1 from aliphatic
                       0.6        2 delta -O from aliphatic
                     -23.7        general corrections
C   154.4          143.0      2-furan
                      13.6        1 -CCCCC
                      -2.2        general corrections
CH  141.5          143.0      2-furan
                      -2.4        1 -CCCCC
                       0.9        general corrections
CH  105.5          109.9      2-furan
                      -5.4        1 -CCCCC
                       1.0        general corrections
CH  110.0          109.9      2-furan
                       0.1        1 -CCCCC
                       0.0        general corrections
CH2 27.4           -11.2      tetrahydrofuran
                      18.2        2 alpha -C from aliphatic
                      18.8        2 beta -C from aliphatic
                      20.2        2 beta -O from aliphatic
                      -2.6        1 gamma -1:C*C*C*C*C*C*1 from aliphatic
                      -2.5        1 gamma -C from aliphatic
                     -12.4        2 gamma -O from aliphatic
                       0.3        1 delta -1:C*C*C*C*C*C*1 from aliphatic
                      -0.3        1 delta -1:C-O-C=C-C=1 from aliphatic
                      -1.1        general corrections
CH2 29.2           -11.2      tetrahydrofuran
                      18.2        2 alpha -C from aliphatic
                      18.8        2 beta -C from aliphatic
                      20.2        2 beta -O from aliphatic
                      -2.6        1 gamma -1:C*C*C*C*C*C*1 from aliphatic
                      -5.0        1 gamma -1:C-O-C=C-C=1 from aliphatic
                      -2.5        1 gamma -C from aliphatic
                      -6.2        1 gamma -O from aliphatic
                       0.3        1 delta -1:C*C*C*C*C*C*1 from aliphatic
                       0.3        1 delta -O from aliphatic
                      -1.1        general corrections
C   145.5          128.5      1-benzene
                      30.3        1 -OCC
                     -14.4        1 -O-C
                      -1.1        1 -C-O
                       2.2        general corrections
C   145.5          128.5      1-benzene
                      30.3        1 -OCC
                     -14.4        1 -O-C
                      -1.1        1 -C-O
                       2.2        general corrections
C   150.1          128.5      1-benzene
                     -14.3        1 -OCC
                      33.5        1 -O-C
                       0.2        1 -C-O
                       2.2        general corrections
C   150.1          128.5      1-benzene
                     -14.3        1 -OCC
                      33.5        1 -O-C
                       0.2        1 -C-O
                       2.2        general corrections
C   120.7          128.5      1-benzene
                      -8.4        1 -OCC
                       1.0        1 -O-C
                      12.4        1 -C-O
                     -12.8        general corrections
C   120.7          128.5      1-benzene
                      -8.4        1 -OCC
                       1.0        1 -O-C
                      12.4        1 -C-O
                     -12.8        general corrections
CH  113.8          128.5      1-benzene
                       0.6        1 -OCC
                     -14.4        1 -O-C
                      -1.2        1 -C-O
                       0.3        general corrections
CH  112.7          128.5      1-benzene
                     -14.3        1 -OCC
                       1.0        1 -O-C
                       0.2        1 -C-O
                      -2.7        general corrections
CH  113.8          128.5      1-benzene
                       0.6        1 -OCC
                     -14.4        1 -O-C
                      -1.2        1 -C-O
                       0.3        general corrections
CH  117.3          128.5      1-benzene
                       0.6        1 -OCC
                      -7.7        1 -O-C
                      -1.2        1 -C-O
                      -2.9        general corrections
CH  112.7          128.5      1-benzene
                     -14.3        1 -OCC
                       1.0        1 -O-C
                       0.2        1 -C-O
                      -2.7        general corrections
CH  117.3          128.5      1-benzene
                       0.6        1 -OCC
                      -7.7        1 -O-C
                      -1.2        1 -C-O
                      -2.9        general corrections
CH2 67.7            -2.3      aliphatic
                       9.1        1 alpha -C
                      49.0        1 alpha -O
                       9.4        1 beta -C
                      20.2        2 beta -O
                      -2.6        1 gamma -1:C*C*C*C*C*C*1
                      -5.0        2 gamma -C
                       0.3        1 delta -C
                       0.3        1 delta -O
                     -10.7        general corrections
CH2 65.0            -2.3      aliphatic
                      24.3        1 alpha -1:C*C*C*C*C*C*1
                      49.0        1 alpha -O
                       0.3        1 delta -O
                      -6.3        general corrections
CH2 65.0            -2.3      aliphatic
                      24.3        1 alpha -1:C*C*C*C*C*C*1
                      49.0        1 alpha -O
                       0.3        1 delta -O
                      -6.3        general corrections
CH3 56.1            -2.3      aliphatic
                      49.0        1 alpha -O
                       9.3        1 beta -1:C*C*C*C*C*C*1
                       0.3        1 delta -O
                      -0.2        general corrections
CH3 56.1            -2.3      aliphatic
                      49.0        1 alpha -O
                       9.3        1 beta -1:C*C*C*C*C*C*1
                       0.3        1 delta -O
                      -0.2        general corrections
CH2 40.4            -2.3      aliphatic
                      17.0        1 alpha -1:C-O-C=C-C=1
                       9.1        1 alpha -C
                       9.4        1 beta -C
                      20.2        2 beta -O
                      -2.6        1 gamma -1:C*C*C*C*C*C*1
                      -5.0        2 gamma -C
                       0.3        1 delta -C
                       0.3        1 delta -O
                      -6.0        general corrections
```

Figure S8. calculated NMR spectra of model **M8**

ChemNMR ^{1}H Estimation

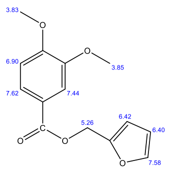

Estimation quality is indicated by color: good, medium, rough

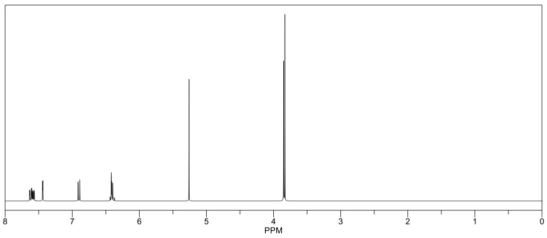


```
Protocol of the H-1 NMR Prediction (Lib=SU Solvent=DMSO 300 MHz):

Node     Shift    Base + Inc.   Comment (ppm rel. to TMS)

CH  7.58             7.38       2-furan
                    -0.08          1 -C-O
                     0.28          general corrections
CH  6.42             6.30       2-furan
                    -0.11          1 -C-O
                     0.23          general corrections
CH  6.40             6.30       2-furan
                    -0.05          1 -C-O
                     0.15          general corrections
CH  7.44             7.26       1-benzene
                    -0.38          1 -O-C
                     0.00          1 -O-C
                     0.71          1 -C(=O)OC
                    -0.15          general corrections
CH  6.90             7.26       1-benzene
                     0.00          1 -O-C
                    -0.38          1 -O-C
                     0.11          1 -C(=O)OC
                    -0.09          general corrections
CH  7.62             7.26       1-benzene
                    -0.32          1 -O-C
                     0.00          1 -O-C
                     0.71          1 -C(=O)OC
                    -0.03          general corrections
CH2 5.26             1.37       methylene
                     1.07          1 alpha -1:C-O-C=C-C=1
                     2.92          1 alpha -OC(=O)-1:C*C*C*C*C*C*1
                    -0.10          general corrections
CH3 3.85             0.86       methyl
                     2.87          1 alpha -O-1:C*C*C*C*C*C*1
                     0.12          general corrections
CH3 3.83             0.86       methyl
                     2.87          1 alpha -O-1:C*C*C*C*C*C*1
                     0.10          general corrections

1H NMR Coupling Constant Prediction

shift   atom index  coupling partner, constant and vector

7.58       16
                  17   7.5  H-C*C-H
                  18   1.5  H-C*CH*C-H
6.42       18
                  17   7.5  H-C*C-H
                  16   1.5  H-C*CH*C-H
6.40       17
                  16   7.5  H-C*C-H
                  18   7.5  H-C*C-H
7.44        5
                   3   1.5  H-C*C*C-H
6.90        2
                   3   7.5  H-C*C-H
7.62        3
                   2   7.5  H-C*C-H
                   5   1.5  H-C*C*C-H
5.26       13
3.85       10
3.83       19
```

ChemNMR ^{13}C Estimation

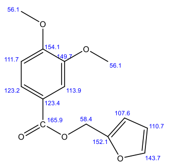

Estimation quality is indicated by color: good, medium, rough

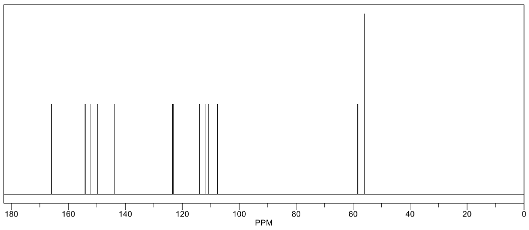


```
Protocol of the C-13 NMR Prediction: (Lib=S)

Node     Shift    Base + Inc.   Comment (ppm rel. to TMS)

C   152.1          143.0      2-furan
                    11.2         1 -C-O
                    -2.1         general corrections
CH  143.7          143.0      2-furan
                    -0.2         1 -C-O
                     0.9         general corrections
CH  107.6          109.9      2-furan
                    -2.0         1 -C-O
                    -0.3         general corrections
CH  110.7          109.9      2-furan
                     0.8         1 -C-O
                     0.0         general corrections
C   149.7          128.5      1-benzene
                    33.5         1 -O-C
                   -14.4         1 -O-C
                    -0.1         1 -C(=O)-O-C
                     2.2         general corrections
C   154.1          128.5      1-benzene
                   -14.4         1 -O-C
                    33.5         1 -O-C
                     4.3         1 -C(=O)-O-C
                     2.2         general corrections
C   123.4          128.5      1-benzene
                     1.0         1 -O-C
                    -7.7         1 -O-C
                     2.0         1 -C(=O)-O-C
                    -0.4         general corrections
CH  113.9          128.5      1-benzene
                   -14.4         1 -O-C
                     1.0         1 -O-C
                     1.2         1 -C(=O)-O-C
                    -2.4         general corrections
CH  111.7          128.5      1-benzene
                     1.0         1 -O-C
                   -14.4         1 -O-C
                    -0.1         1 -C(=O)-O-C
                    -3.3         general corrections
CH  123.2          128.5      1-benzene
                    -7.7         1 -O-C
                     1.0         1 -O-C
                     1.2         1 -C(=O)-O-C
                     0.2         general corrections
C   165.9          166.0      1-carboxyl
                     6.0         1 -1:C*C*C*C*C*C*1
                    -5.0         1 -C from O-carboxyl
                    -1.1         general corrections
CH2 58.4            -2.3      aliphatic
                    17.0         1 alpha -1:C-O-C=C-C=1
                    54.9         1 alpha -O-C=O
                    -2.6         1 gamma -1:C*C*C*C*C*C*1
                    -8.6         general corrections
CH3 56.1            -2.3      aliphatic
                    49.0         1 alpha -O
                     9.3         1 beta -1:C*C*C*C*C*C*1
                     0.3         1 delta -O
                    -0.2         general corrections
CH3 56.1            -2.3      aliphatic
                    49.0         1 alpha -O
                     9.3         1 beta -1:C*C*C*C*C*C*1
                     0.3         1 delta -O
                    -0.2         general corrections
```

Figure S9. calculated NMR spectra of model **M9**

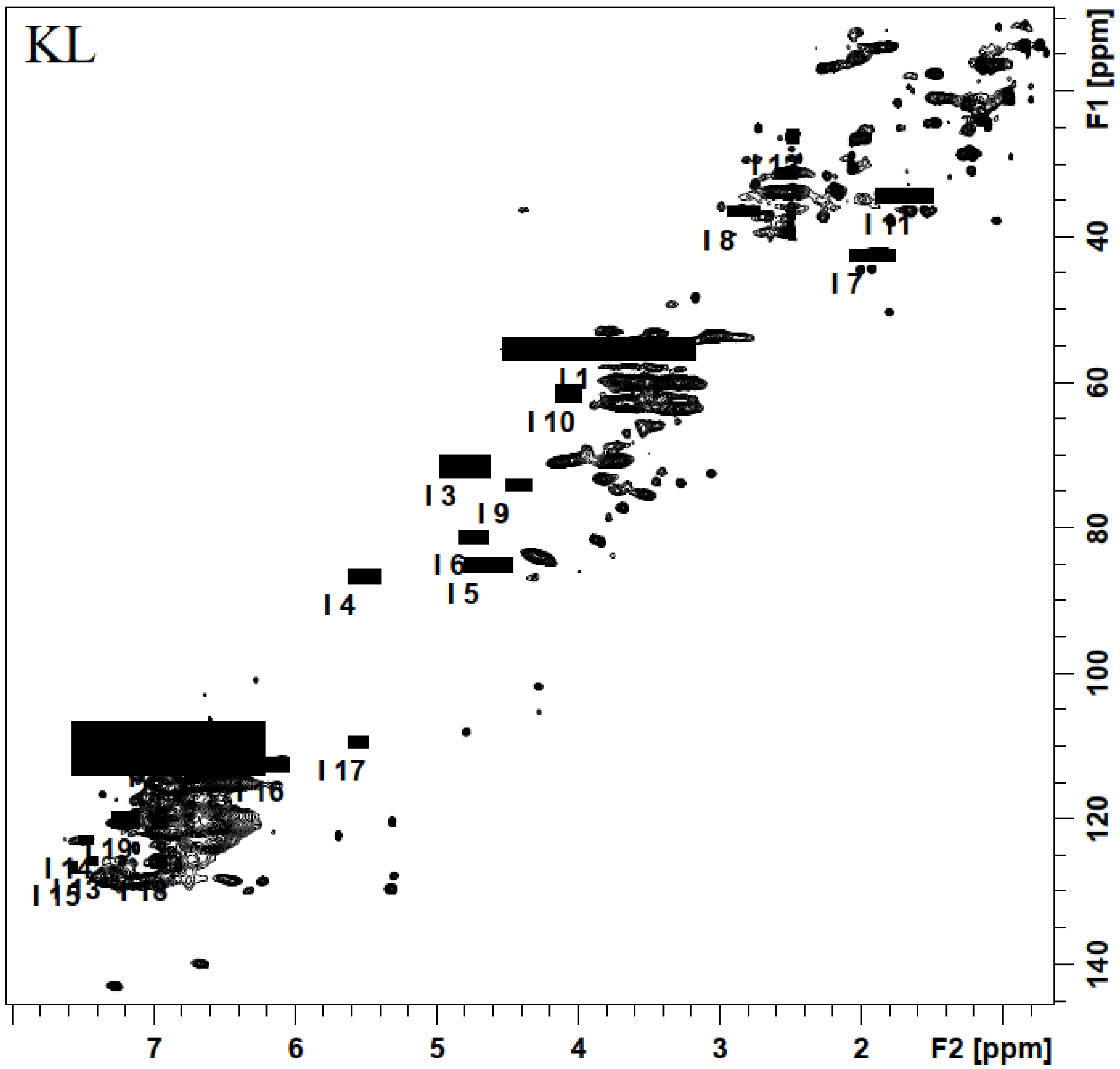

Figure S10. integral of typical structures in KL (nlev = 50, lev0 = 20, toplev = 100 %)

Detailed data set for Figure S10:

Name = KL xzc12-4 ExpNo = 2 ProcNo = 1

Baseline = 0 Noise = 0

# SI_F1 row1 row2 row1(ppm) row2(ppm) abs. Int. Integral norm. Int. Mode

SI_F2 col1 col2 col1(ppm) col2(ppm)

Methoxyl (I1) 1024 739 754 56.87234 53.74060 7.4108e+13 7.0675e+07 300 a

1024 578 706 4.53892 3.17355

G2(I2) 1024 468 503 113.83346 106.57680 2.2984e+13 2.1919e+07 93.043 a

1024 294 421 7.57623 6.21993

Aα(I3) 1024 663 677 72.88113 70.01336 1.7135e+12 1.6341e+06 6.9364 a

1024 537 570 4.98152 4.62657

**Bα(I4)**	**1024**	**593**	**603**	**87.71448**	**85.63781**	**5.2104e+11**	**4.969e+05**	**2.1092**	**a**
	**1024**	**476**	**498**	**5.63634**	**5.39155**				
**Cα(I5)**	**1024**	**601**	**610**	**86.03336**	**84.05559**	**8.9228e+11**	**8.5095e+05**	**3.6121**	**a**
	**1024**	**553**	**584**	**4.80405**	**4.47970**				
**C'α(I6)**	**1024**	**620**	**628**	**81.97892**	**80.19892**	**3.2991e+11**	**3.1463e+05**	**1.3355**	**a**
	**1024**	**550**	**569**	**4.84076**	**4.63881**				
**SRβ(I7)**	**1024**	**804**	**812**	**43.21573**	**41.53674**	**1.2648e+12**	**1.2062e+06**	**5.1202**	**a**
	**1024**	**807**	**837**	**2.08733**	**1.76134**				
**Arα(I8)**	**1024**	**834**	**840**	**37.01077**	**35.76978**	**3.9495e+11**	**3.7666e+05**	**1.5988**	**a**
	**1024**	**727**	**748**	**2.94367**	**2.71499**				
**AGα(I9)**	**1024**	**654**	**662**	**74.79440**	**73.24104**	**2.9683e+11**	**2.8307e+05**	**1.2016**	**a**
	**1024**	**580**	**597**	**4.51274**	**4.33490**				
**Dγ(I10)**	**1024**	**712**	**723**	**62.53218**	**60.29508**	**7.1084e+11**	**6.7791e+05**	**2.8776**	**a**
	**1024**	**613**	**630**	**4.16823**	**3.97984**				
**DHCAβ(I11)**	**1024**	**842**	**852**	**35.36743**	**33.23687**	**2.0074e+12**	**1.9144e+06**	**8.1264**	**a**
	**1024**	**825**	**863**	**1.89965**	**1.48361**				
**AVβ(I12)**	**1024**	**881**	**890**	**27.05822**	**25.14071**	**2.3597e+11**	**2.2504e+05**	**0.95525**	**a**
	**1024**	**766**	**773**	**2.52763**	**2.44913**				
**V6(I13)**	**1024**	**408**	**416**	**126.53445**	**124.84582**	**2.1618e+11**	**2.0616e+05**	**0.87512**	**a**
	**1024**	**302**	**313**	**7.49588**	**7.37565**				
**VA6(I14)**	**1024**	**423**	**428**	**123.44588**	**122.30872**	**1.6251e+11**	**1.5498e+05**	**0.65786**	**a**
	**1024**	**298**	**307**	**7.53416**	**7.43899**				
**Bi6(I16)**	**1024**	**405**	**411**	**127.04691**	**125.78339**	**1.6579e+11**	**1.5811e+05**	**0.67115**	**a**
	**1024**	**292**	**297**	**7.60157**	**7.55002**				
**E-EEα(I17)**	**1024**	**471**	**480**	**113.33772**	**111.31609**	**5.9059e+11**	**5.6323e+05**	**2.3908**	**a**
	**1024**	**421**	**437**	**6.21767**	**6.05112**				
**Z-EEα(I18)**	**1024**	**486**	**493**	**110.05257**	**108.59953**	**2.5104e+11**	**2.3941e+05**	**1.0162**	**a**
	**1024**	**477**	**489**	**5.61890**	**5.49201**				
**SB1α(I19)**	**1024**	**407**	**416**	**126.73103**	**124.83575**	**7.7581e+11**	**7.3987e+05**	**3.1406**	**a**
	**1024**	**349**	**356**	**6.99091**	**6.91557**				
**SB5β(I20)**	**1024**	**433**	**444**	**121.17155**	**118.96039**	**1.3081e+12**	**1.2475e+06**	**5.2952**	**a**
	**1024**	**320**	**338**	**7.30020**	**7.10987**				

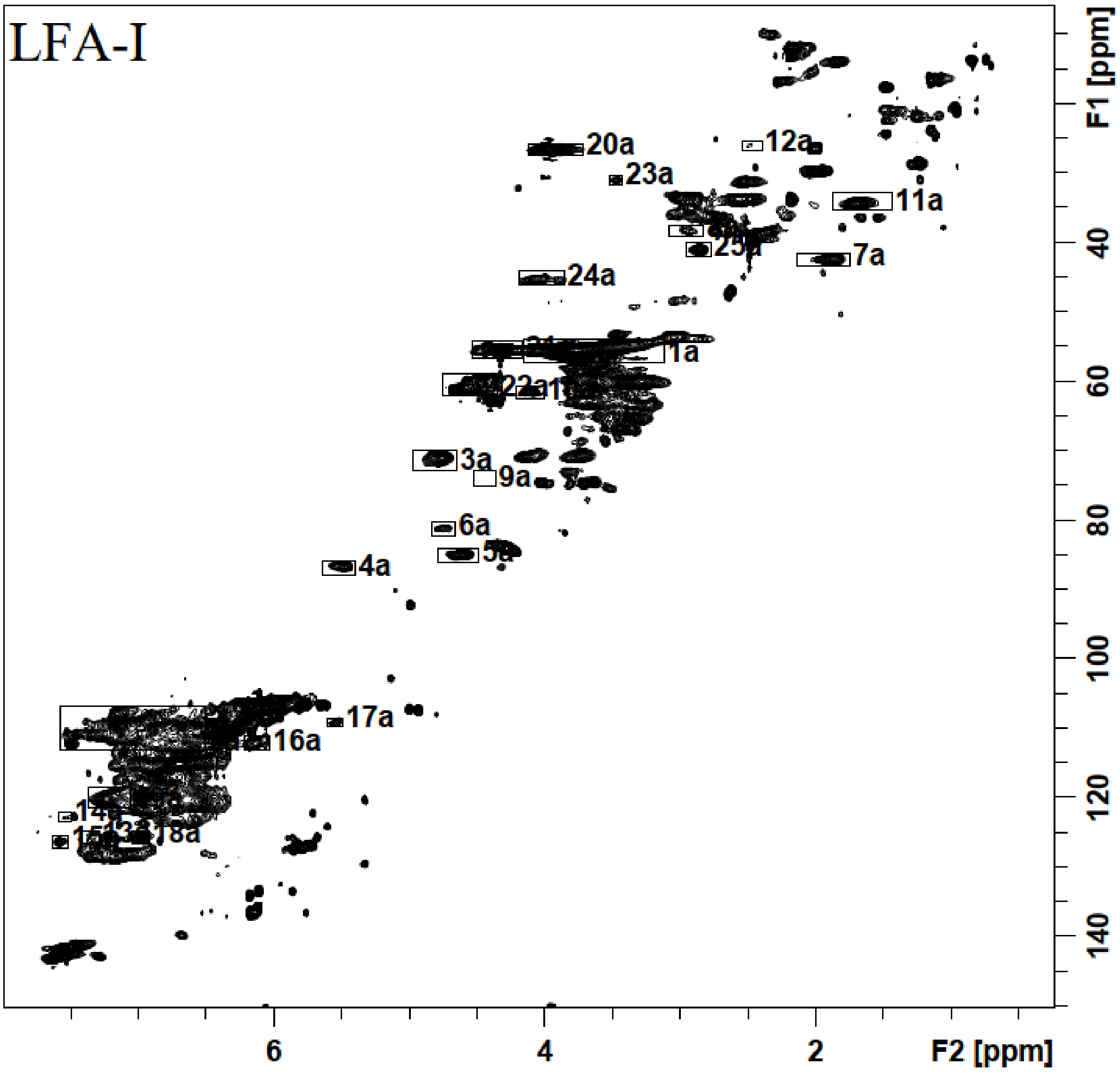

Figure S11. integral of typical aromatic units in LFA-I (nlev = 50, lev0 = 20, toplev = 100 %)

Detailed data set for Figure S11:

Name = LFA-I xzc33-S ExpNo = 2 ProcNo = 1

Baseline = 0 Noise = 0

# SI_F1 row1 row2 row1(ppm) row2(ppm) abs. Int. Integral norm. Int. Mode

SI_F2 col1 col2 col1(ppm) col2(ppm)

Methoxyl(I1) 1024 738 753 57.07990 54.00709 5.9417e+13 5.6665e+07 300 a

1024 614 710 4.15006 3.12031

G2(I2) 1024 472 501 113.11025 106.89172 1.8077e+13 1.7239e+07 91.27 a

1024 294 393 7.57550 6.51197

Aα(I3) 1024 664 677 72.67445 70.06174 1.2703e+12 1.2115e+06 6.414 a

1024 537 567 4.96677 4.65019

Bα(I4) 1024 592 601 87.80064 85.94424 4.7693e+11 4.5483e+05 2.408 a

1024 475 497 5.63108 5.39754

Cα(I5) 1024 601 609 85.94424 84.22535 7.5991e+11 7.2471e+05 3.8368 a

1024 555 582 4.77474 4.49449

C’α(I6) 1024 619 627 82.09393 80.51256 2.6982e+11 2.5732e+05 1.3624 a

1024 550 566 4.83183 4.66576

SRβ(I7) 1024 804 812 43.29043 41.63521 9.814e+11 9.3594e+05 4.9551 a

1024 802 838 2.13209 1.75063

Arα(I8) 1024 824 831 39.02505 37.68814 3.7038e+11 3.5322e+05 1.87 a

1024 714 737 3.07212 2.83371

AGα(I9) 1024 653 663 75.12163 73.01823 2.1066e+11 2.009e+05 1.0636 a

1024 579 594 4.51650 4.36076

Dγ(I10) 1024 713 721 62.42607 60.77340 4.6521e+11 4.4366e+05 2.3489 a

1024 609 627 4.19903 4.00735

DHCAβ(I11) 1024 842 854 35.33900 32.87951 1.6852e+12 1.6071e+06 8.5086 a

1024 821 867 1.92651 1.43658

AVβ(I12) 1024 883 889 26.74288 25.40851 1.2242e+11 1.1675e+05 0.6181 a

1024 765 777 2.53260 2.40511

V6(I13) 1024 409 415 126.34295 125.13434 1.8565e+11 1.7705e+05 0.93733 a

1024 309 320 7.41127 7.29067

VA6(I14) 1024 423 428 123.42866 122.40794 9.5628e+10 91198 0.48283 a

1024 293 301 7.58153 7.49676

Bi6(I15) 1024 405 412 127.11896 125.62713 1.9833e+11 1.8914e+05 1.0014 a

1024 289 299 7.62921 7.51795

E-EEα(I16) 1024 472 481 113.06442 111.18002 6.0827e+11 5.8009e+05 3.0712 a

1024 421 438 6.21463 6.02920

Z-EEα(I17) 1024 488 493 109.76671 108.58895 1.5602e+11 1.4879e+05 0.78776 a

1024 478 488 5.60535 5.49939

SB1α(I18) 1024 408 416 126.49082 124.92048 5.5834e+11 5.3247e+05 2.8191 a

1024 344 355 7.03583 6.91927

SB5β(I19) 1024 433 445 121.18516 118.70278 1.2164e+12 1.1601e+06 6.1417 a

1024 305 332 7.45604 7.16186

CH2 in pFA(I20) 1024 880 887 27.37992 25.91314 1.4712e+13 1.403e+07 74.28 a

1024 617 654 4.11856 3.72374

CH2OH in pFA(I21) 1024 741 752 56.59333 54.14869 8.2265e+12 7.8454e+06 41.536 a

1024 578 612 4.52887 4.17275

CH2OCH2 in pFA(I22) 1024 715 729 61.97152 59.16019 4.1691e+12 3.9759e+06 21.05 a

1024 558 595 4.74564 4.35081

New peak 2(I23) 1024 860 865 31.53580 30.43572 1.1151e+11 1.0635e+05 0.56303 a

1024 673 681 3.51471 3.42955

New peak 3(I24) 1024 791 800 45.95916 44.24791 5.7779e+11 5.5102e+05 2.9173 a

1024 611 641 4.18050 3.85535

New peak 4(I25) 1024 811 819 41.92551 40.09203 4.84e+11 4.6157e+05 2.4437 a

1024 726 742 2.94957 2.77926

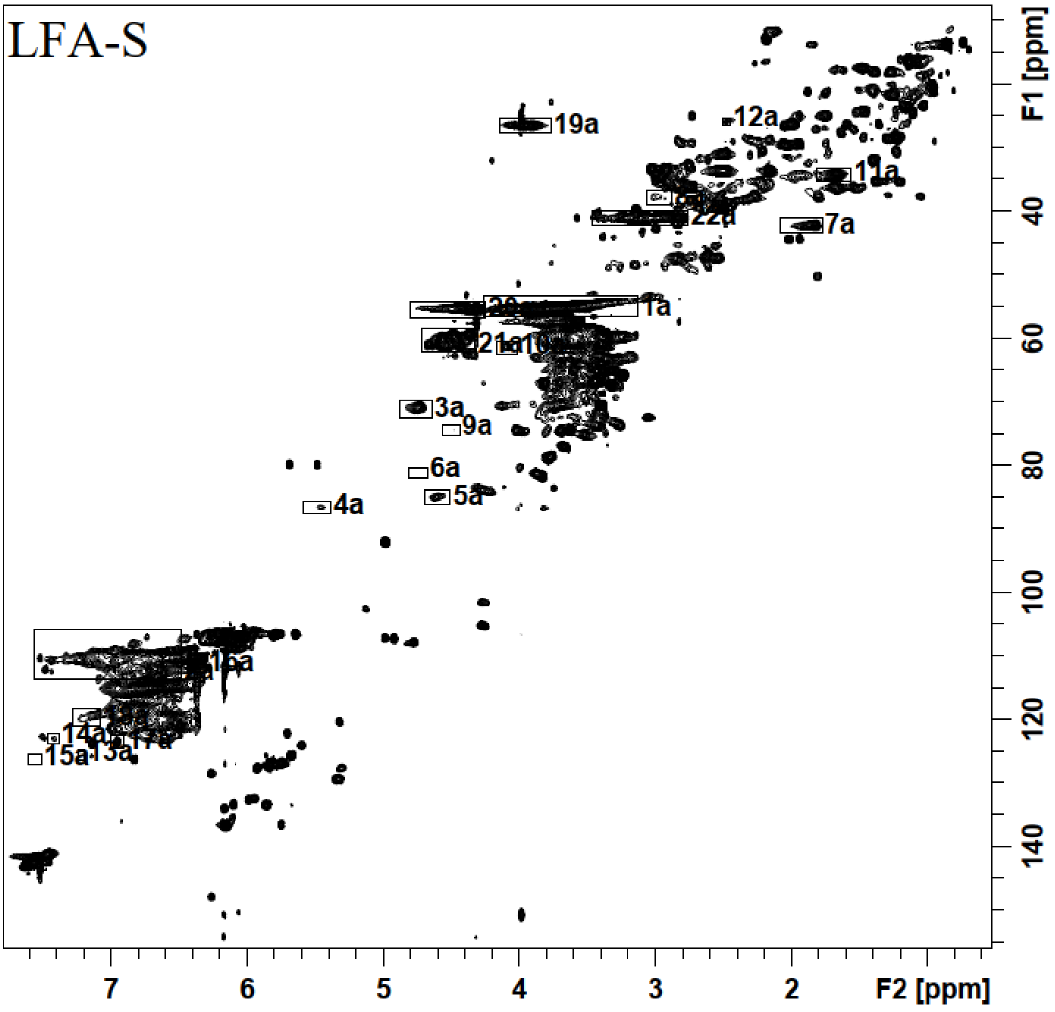

Figure S12. integral of typical aromatic units in LFA-S (nlev = 50, lev0 = 20, toplev = 100 %)

Detailed data set for Figure S12:

Name = LFA-S xzc33-L ExpNo = 2 ProcNo = 1

Baseline = 0 Noise = 0

# SI_F1 row1 row2 row1(ppm) row2(ppm) abs. Int. Integral norm. Int. Mode

SI_F2 col1 col2 col1(ppm) col2(ppm)

**Methoxyl(I1)**	**1024**	**741**	**755**	**56.33739**	**53.46749**	**3.4298e+13**	**3.271e+07**	**300**	**a**
	**1024**	**604**	**709**	**4.24803**	**3.12895**				
**G2(I2)**	**1024**	**472**	**497**	**112.91586**	**107.64885**	**1.0506e+13**	**1.0019e+07**	**91.892**	**a**
	**1024**	**296**	**384**	**7.54531**	**6.60333**				
**Aα(I3)**	**1024**	**665**	**677**	**72.34975**	**69.77652**	**6.9156e+11**	**6.5953e+05**	**6.0489**	**a**
	**1024**	**546**	**567**	**4.87054**	**4.64776**				
**Bα(I4)**	**1024**	**592**	**601**	**87.63552**	**85.69382**	**2.2353e+11**	**2.1317e+05**	**1.9551**	**a**
	**1024**	**477**	**499**	**5.61500**	**5.37349**				
**Cα(I5)**	**1024**	**600**	**611**	**86.08216**	**83.62267**	**4.1166e+11**	**3.9259e+05**	**3.6007**	**a**
	**1024**	**553**	**583**	**4.79992**	**4.47539**				
**C’α(I6)**	**1024**	**618**	**626**	**82.14528**	**80.59537**	**1.487e+11**	**1.4181e+05**	**1.3006**	**a**
	**1024**	**552**	**568**	**4.80493**	**4.63447**				
**SRβ(I7)**	**1024**	**804**	**814**	**43.11005**	**41.08134**	**7.1584e+11**	**6.8268e+05**	**6.2613**	**a**
	**1024**	**816**	**834**	**1.98494**	**1.78934**				
**Arα(I8)**	**1024**	**825**	**832**	**38.70461**	**37.15125**	**2.5115e+11**	**2.3951e+05**	**2.1967**	**a**
	**1024**	**716**	**729**	**3.04900**	**2.91316**				
**AGα(I9)**	**1024**	**652**	**658**	**75.17170**	**73.89760**	**1.0919e+11**	**1.0413e+05**	**0.95505**	**a**
	**1024**	**575**	**586**	**4.55936**	**4.43995**				
**Dγ(I10)**	**1024**	**712**	**721**	**62.36367**	**60.55312**	**2.6153e+11**	**2.4941e+05**	**2.2875**	**a**
	**1024**	**613**	**626**	**4.15770**	**4.02201**				
**DHCAβ(I11)**	**1024**	**843**	**850**	**34.99521**	**33.37225**	**9.0064e+11**	**8.5892e+05**	**7.8777**	**a**
	**1024**	**834**	**854**	**1.78934**	**1.57925**				
**AVβ(I12)**	**1024**	**885**	**890**	**26.14826**	**25.11269**	**6.6369e+10**	**63295**	**0.58051**	**a**
	**1024**	**771**	**776**	**2.46033**	**2.41505**				

V6(I13) 1024 408 415 126.43388 124.90428 1.0777e+11 1.0277e+05 0.9426 a

1024 322 330 7.27053 7.18673

VA6(I14) 1024 422 427 123.45518 122.28786 8.1289e+10 77524 0.71102 a

1024 305 311 7.45824 7.38785

Bi6(I15) 1024 405 412 126.89611 125.41914 9.0653e+10 86453 0.79292 a

1024 291 299 7.59788 7.51748

E-EEα(I16) 1024 475 479 112.34071 111.42860 3.8079e+11 3.6315e+05 3.3307 a

1024 400 413 6.43551 6.29877

SB1α(I17) 1024 417 427 124.42645 122.44919 3.2148e+11 3.0659e+05 2.8119 a

1024 345 356 7.01968 6.91093

SB5β(I18) 1024 430 444 121.77834 118.70651 6.6448e+11 6.337e+05 5.8121 a

1024 320 341 7.29651 7.06912

CH2 in pFA(I19) 1024 878 888 27.48687 25.41445 1.7224e+13 1.6426e+07 150.66 a

1024 615 649 4.13779 3.76692

CH2OH in pFA(I20) 1024 740 751 56.50079 54.21022 2.1092e+13 2.0115e+07 184.49 a

1024 553 604 4.80005 4.25434

CH2OCH2 in pFA(I21) 1024 714 731 62.06361 58.57322 5.8616e+12 5.5901e+06 51.27 a

1024 561 597 4.70998 4.32852

New peak 7(I22) 1024 809 819 41.99383 40.03048 1.1442e+13 1.0912e+07 100.08 a

1024 678 743 3.45964 2.76029
